# Factor quinolinone inhibitors disrupt spindles and multiple LSF (TFCP2)-protein interactions in mitosis, including with microtubule-associated proteins

**DOI:** 10.1371/journal.pone.0268857

**Published:** 2022-06-15

**Authors:** Sarah A. Yunes, Jennifer L. S. Willoughby, Julian H. Kwan, Jessica M. Biagi, Niranjana Pokharel, Hang Gyeong Chin, Emily A. York, Kuan-Chung Su, Kelly George, Jagesh V. Shah, Andrew Emili, Scott E. Schaus, Ulla Hansen

**Affiliations:** 1 Department of Biology, Boston University, Boston, Massachusetts, United States of America; 2 Program in Molecular Biology, Cell Biology, and Biochemistry, Boston University, Boston, Massachusetts, United States of America; 3 Alnylam Pharmaceuticals, Cambridge, Massachusetts, United States of America; 4 Department of Biochemistry and Center for Network Systems Biology, Boston University School of Medicine, Boston, Massachusetts, United States of America; 5 Department of Chemistry and Center for Molecular Discovery, Boston University, Boston, Massachusetts, United States of America; 6 New England Biolabs, Ipswich, Massachusetts, United States of America; 7 Whitehead Institute for Biomedical Research, Cambridge, Massachusetts, United States of America; 8 Department of Systems Biology, Harvard Medical School, Boston, Massachusetts, United States of America; Centre de Recherche en Biologie cellulaire de Montpellier, FRANCE

## Abstract

Factor quinolinone inhibitors (FQIs), a first-in-class set of small molecule inhibitors targeted to the transcription factor LSF (TFCP2), exhibit promising cancer chemotherapeutic properties. FQI1, the initial lead compound identified, unexpectedly induced a concentration-dependent delay in mitotic progression. Here, we show that FQI1 can rapidly and reversibly lead to mitotic arrest, even when added directly to mitotic cells, implying that FQI1-mediated mitotic defects are not transcriptionally based. Furthermore, treatment with FQIs resulted in a striking, concentration-dependent diminishment of spindle microtubules, accompanied by a concentration-dependent increase in multi-aster formation. Aberrant γ-tubulin localization was also observed. These phenotypes suggest that perturbation of spindle microtubules is the primary event leading to the mitotic delays upon FQI1 treatment. Previously, FQIs were shown to specifically inhibit not only LSF DNA-binding activity, which requires LSF oligomerization to tetramers, but also other specific LSF-protein interactions. Other transcription factors participate in mitosis through non-transcriptional means, and we recently reported that LSF directly binds α-tubulin and is present in purified cellular tubulin preparations. Consistent with a microtubule role for LSF, here we show that LSF enhanced the rate of tubulin polymerization *in vitro*, and FQI1 inhibited such polymerization. To probe whether the FQI1-mediated spindle abnormalities could result from inhibition of mitotic LSF-protein interactions, mass spectrometry was performed using as bait an inducible, tagged form of LSF that is biotinylated by endogenous enzymes. The global proteomics analysis yielded expected associations for a transcription factor, notably with RNA processing machinery, but also to nontranscriptional components. In particular, and consistent with spindle disruption due to FQI treatment, mitotic, FQI1-sensitive interactions were identified between the biotinylated LSF and microtubule-associated proteins that regulate spindle assembly, positioning, and dynamics, as well as centrosome-associated proteins. Probing the mitotic LSF interactome using small molecule inhibitors therefore supported a non-transcriptional role for LSF in mediating progression through mitosis.

## Introduction

The class of small molecules named Factor Quinolinone Inhibitors (FQIs) exhibit promising anti-cancer properties. FQIs were initially isolated in a screen for specific DNA-binding inhibitors of the transcription factor LSF (encoded by *TFCP2*, Gene ID: 7024) [1]. LSF expression is elevated in a number of cancers, and was first shown to promote oncogenesis in hepatocellular carcinoma [2]. Subsequently, LSF has been linked to increased proliferation, migration, and tumorigenesis in a number of cancer types [3]. FQIs effectively reduce cell proliferation in multiple cancer cell lines and blunt tumor progression in rodent tumor models, with minimal detectable side effects [1, 4, 5]. At the cellular level, we previously showed that FQIs unexpectedly interfered with mitotic progression, causing an arrest with condensed but unaligned chromosomes. Furthermore, knockdown of LSF using an LSF-specific siRNA in HeLa cells showed mitotic defects analogous to those of FQI1, the initial compound identified, suggesting that FQI1-mediated lack of mitotic chromosomal alignment was related to LSF functionality [6].

Mitosis involves a complex progression of temporally and spatially regulated protein-protein interactions. Key early steps that drive the process are the concurrent establishment of the bipolar spindle and of chromosomal alignment. Formation of the mitotic spindle requires rapid dissolution of the interphase network of microtubules and reassembly into the bipolar structure generally anchored by a pair of centrosomes. Alterations in microtubule structures and dynamics are driven by mitotic microtubule associated proteins and by post-translational modifications of both the α/β-tubulin structural components and their associated proteins [7–9]. Given the complexity of the mitotic spindle and the speed of its transitions during mitosis, both the proteins and mechanisms that regulate such spindle dynamics are yet to be fully understood.

Cancer cells are vulnerable to a variety of microtubule inhibitors that directly bind tubulins, which often also affect not only mitotic, but also interphase microtubules [10]. Several such compounds are efficacious chemotherapeutic agents for treating specific cancers, although a limitation to direct microtubule-binding inhibitors can be their significant side effects [11]. Here we demonstrate that FQI treatment also results in disruption of the mitotic spindle. The known target of FQI1, LSF, directly and specifically binds α-tubulin *in vitro* and was identified in purified cellular tubulin preparations [12], and siRNA against LSF also induces mitotic defects [6]. Furthermore, multiple other transcription factors moonlight to promote critical mitotic structures [13]. Thus, we pursued the hypothesis that the FQI1-driven mitotic defects result from the inhibition of LSF, rather than from direct interaction with tubulins. Investigation of the window in the cell cycle in which FQI1 exerted mitotic consequences led to the conclusion that the mechanism did not result from transcriptional dysregulation. However, proteomics analysis identified mitotic LSF-protein interactions that were disrupted in the presence of FQI1, including associations with other microtubule associated proteins that contribute to spindle formation and dynamics. The unanticipated dual mode of action of FQIs–the described inhibition of LSF transcriptional activity as previously demonstrated, plus non-transcriptional microtubule effects described here, may provide the mechanistic underpinnings for why these compounds potently attack cancer cells without apparent toxicity.

## Materials and methods

### Cell culture, synchronization, and treatments

HeLa cells (from Devanand Sarkar, Virginia Commonwealth University), validated to contain the reported, specific HPV-18 gene segments [6], were cultured in DMEM with 10% fetal bovine serum (FBS). The DLD-1 Flp-In™ T-REx™ TIR1 BioLSF cell line (DLD-1 derived cells) was generated from the DLD-1 Flp-In™ T-REx™ TIR1 (parental DLD-1) cells [14] (gift of Iain Cheeseman) by insertion of a cDNA encoding a constitutively biotinylated domain fused to LSF into the single genomic FRT site (S1 File). DLD-1 cell lines were cultured in DMEM with 10% tetracycline-free FBS, preventing expression of the inserted cDNA. BioLSF expression was induced by addition of 1 μg/mL doxycycline. For maintenance of the genetic makeup, parental and derived DLD-1 cells were periodically propagated in zeocin, blasticidin, and puromycin, or hygromycin, blasticidin, and puromycin, respectively. RPE-hTERT-Flp-In (RPE) cells [15] (provided by Patrick Meraldi, Université de Genève) were cultured in DMEM:F12 1:1 media with 10% FBS. All cells were maintained at 37°C in 5% CO_2_.

HeLa or DLD-1 cells were synchronized using a double 2 mM thymidine block protocol, with 18 or 15–24 hour blocks, respectively, separated by a 6-hour release in media containing 20 μM each thymidine and 2’deoxycytidine. DLD-1 cells were synchronized in mitosis by a single thymidine block and release, as indicated above, followed by addition of 10 μM STLC (Sigma Aldrich) five hours after the G1/S release. Mitotic entry following release from the final thymidine blocks (consistently 7.5 hours for HeLa cells and 9.5–10 hours for DLD-1 cells) was determined by DNA profiling by flow cytometry and visual inspection of cell rounding.

RPE cells were synchronized by serum starvation [16], involving incubation in DMEM:F12 lacking serum for 24–48 hours. After subsequent addition of media plus 10% FBS, cells were consistently mitotic at 24 hours, as determined by cell rounding.

FQI1 was synthesized as previously described [1] and FQI34 was synthesized and characterized as described in S1 File. Both were dissolved in anhydrous DMSO. Final DMSO concentrations in the cell media were maximally 0.5%, and constant across each experiment.

### Cellular DNA profiling by flow cytometry

Non-attached cells from the media and cells trysinized from the dish were combined, fixed with ethanol, and stained with propidium iodide. Samples were analyzed using a FACSCalibur flow cytometer and CellQuest Pro software. Image analysis was performed with FlowJo software, using curve smoothing (Becton Dickinson).

### Immunofluorescence

Cells were grown on poly-L-lysine coated coverslips and fixed with formaldehyde. Primary antibodies included: mouse monoclonal anti-α-tubulin antibody (1:500, Thermo Fisher 62204), mouse monoclonal anti-γ-tubulin (1:1000, Abcam ab11316), rabbit polyclonal anti-α-tubulin (1:5000, Thermo Fisher PA529444), and anti-α-tubulin antibody (Abcam #AB7750). Secondary antibodies included: goat anti-mouse IgG-Texas Red (1:200, Thermo Fisher T-862), goat anti-mouse IgG-Alexa Fluor 546 (1:500, Thermo Fisher A11003), and goat anti-rabbit IgG-Cy5 (1:1000, Thermo Fisher A10523). DNA was stained with Hoechst 33342 or DAPI. For the cold-stable microtubule assays, cells were incubated an ice-water bath for 15 minutes in Leibovitz’s L-15 media without phenol red (Gibco) with 10% FBS. Images were taken using a DeltaVision Core deconvolution microscope equipped with a CoolSnap HQ2 CCD camera using the 60X objective and the 1.5X magnifier setting. Across each cell, at least 40 Z-plane slices were taken 0.2 μm apart. The images were deconvolved and the 20 z-stacks representing the middle of the cell were max projected in z. All other samples were imaged using a Nikon NiE widefield microscope utilizing 40X magnification and a Nikon DS-Qi1Mc camera or a Zeiss Axioimager M1 microscope utilizing 63x and 100x magnifications.

### Tubulin polymerization assays

Quantitative determination of tubulin polymerization *in vitro* was carried out using the *in vitro* polymerization assay kits from either Millipore (17–10194) or Cytoskeleton (BK004P) according to the manufacturer’s conditions. For the Millipore kit, thawed tubulin was mixed with purified His-LSF in 70 μL final volumes of 1X PB-GTP solutions, and the 96-well plate was transferred to the pre-warmed (37°C) SpectraMax M5 Microplate Reader. Tubulin polymerization was followed by measuring turbidity (light scattering) every minute at 350 nm for 1 hour, as per the manufacturer’s instructions. For the Cytoskeleton kit, the inhibitors were added to 4 mg/ml tubulin in a 100-μL final volume of 80 mM PIPES pH 6.9, 0.5 mM EGTA, 2 mM MgCl_2_, 1 mM GTP, and after transfer to 37°C, absorbance was measured every minute at 340 nm for 1 hour, as per the manufacturer’s instructions.

### Mass spectrometry

Mitotic parental DLD-1 and DLD-1 derived cells were treated with 1% formaldehyde for 5 minutes at room temperature, followed by glycine to a final concentration of 125 mM to quench the reaction. Cells collected both from the media and the dishes were lysed in 50 mM HEPES pH 7.5, 250 mM NaCl, 50 mM NaF, 5 mM EDTA, 1% Triton X-100, 1:200 Protease Inhibitor Cocktail (Abcam ab201111), followed by sonication and centrifugation to remove particulates. BioLSF and associated proteins were bound to Dynabeads MyOne Streptavidin C1 beads (Invitrogen), washing with 2% sodium dodecyl sulfate (twice), then with high salt buffer (50 mM HEPES pH 7.5, 500 mM NaCl, 50 mM NaF, 5 mM EDTA, 0.1% sodium deoxycholate, 1% Triton X-100), LiCl buffer (10 mM Tris-Cl pH 8, 250 mM LiCl, 1 mM EDTA, 0.5% Igepal CA-630, 0.5% sodium deoxycholate), and 1X PBS. Proteins were eluted by incubating at 100°C for 5 minutes in 37.5 mM HEPES, 50 mM Tris-Cl, pH 7.5, 188 mM NaCl, 37.5 mM NaF, 3.8 mM EDTA, 1:267 Protease Inhibitor Cocktail (Abcam ab201111), 0.75% Triton X-100, 2% SDS, 10% glycerol, 0.01% bromophenol blue, 1.25% β-mercaptoethanol. Samples were electrophoresed into an acrylamide gel and digested in-gel with trypsin. Resulting peptides were separated by reverse-phase EASY-nLC (Thermo) on a 60 min gradient and analyzed on a Q Exactive HF-X mass spectrometer (Thermo). Data-dependent fragmentation used collision-induced dissociation RAW files were searched using MaxQuant version 1.6.2.2 under standard settings using the UniProt human database. The searches allowed for two missed trypsin cleavage sites, variable modifications for N-terminal acetylation, and methionine oxidation. Ion tolerances of 20 and 4.5 ppm were set for first and second searches, respectively. Candidate peptides and protein identifications were filtered based on a 1% false discovery rate threshold based on searching of the reverse sequence database. The mass spectrometry proteomics data have been deposited to the ProteomeXchange Consortium via the PRIDE [17] partner repository with the dataset identifier PXD024149. Gene ontology analysis was performed using the Database for Annotation, Visualization and Integrated Discovery (DAVID) v6.8 [18, 19].

## Results/Discussion

### FQI1 treatment in mitosis causes a rapid and reversible arrest

**Fig 1:**
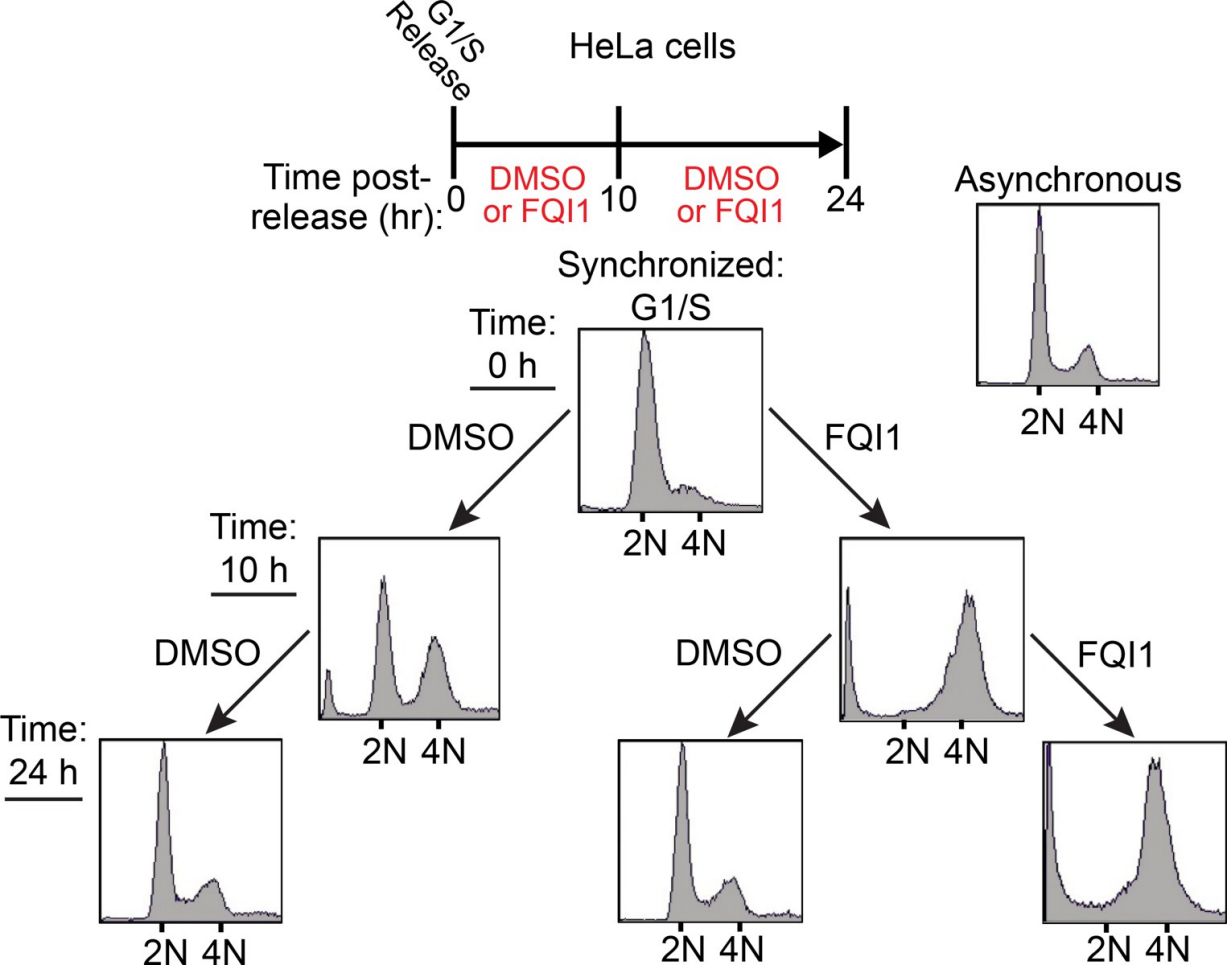
FQI1 treatment induces a rapid and reversible cell cycle arrest with 4N DNA content. Top: Schematic for treatment of HeLa cells following release from a double thymidine block. Cells harvested at 0, 10, and 24 h, as indicated and described in the text, were analyzed for cellular DNA content by propidium iodide staining and flow cytometry. Top right: Asynchronous DNA profile as control.

Since FQI1 can provoke mitotic arrest in multiple cell types [5, 6] and FQI1 inhibits the transcription factor LSF [1, 4, 6, 12, 20], we first hypothesized that the arrest resulted from dysregulated gene expression. If so, the mitotic block should be irreversible, since RNA polymerase II transcription is generally inhibited during mitosis [21–23], which should prevent mitotic recovery from transcriptional defects. Reversibility was tested by incubating synchronized HeLa cells with or without FQI1 between G1/S and mitosis, followed by its removal or maintenance for another 14 hours (Fig 1). By measuring cell cycle progression with cellular DNA profiling, FQI1-treated cells demonstrated a mitotic arrest (4N DNA), whereas a significant proportion of the control cells divided to re-enter G1. With continued incubation with FQI1, the majority of cells remained with 4N DNA, although some exhibited sub-G1 DNA (Fig 1), consistent with FQI1-induced apoptosis [1, 6, 12, 20]. In contrast, when FQI1 was removed, cells completed mitosis, reentering G1. The reversibility of the FQI1-mediated arrest was particularly unexpected because cells were exposed to FQI1 throughout S and G2, the time frame during which key mitotic genes are upregulated [24–27]. To test whether FQI1 was required during S and G2 in order to block mitotic progression, FQI1 was instead added roughly 30 minutes prior to mitotic entry of synchronized HeLa cells. Such limited incubation also sufficed to inhibit cell division (panel A in S1 Fig), suggesting that FQI1 interferes with processes occurring directly in mitosis, rather than in S or G2.

**Fig 2:**
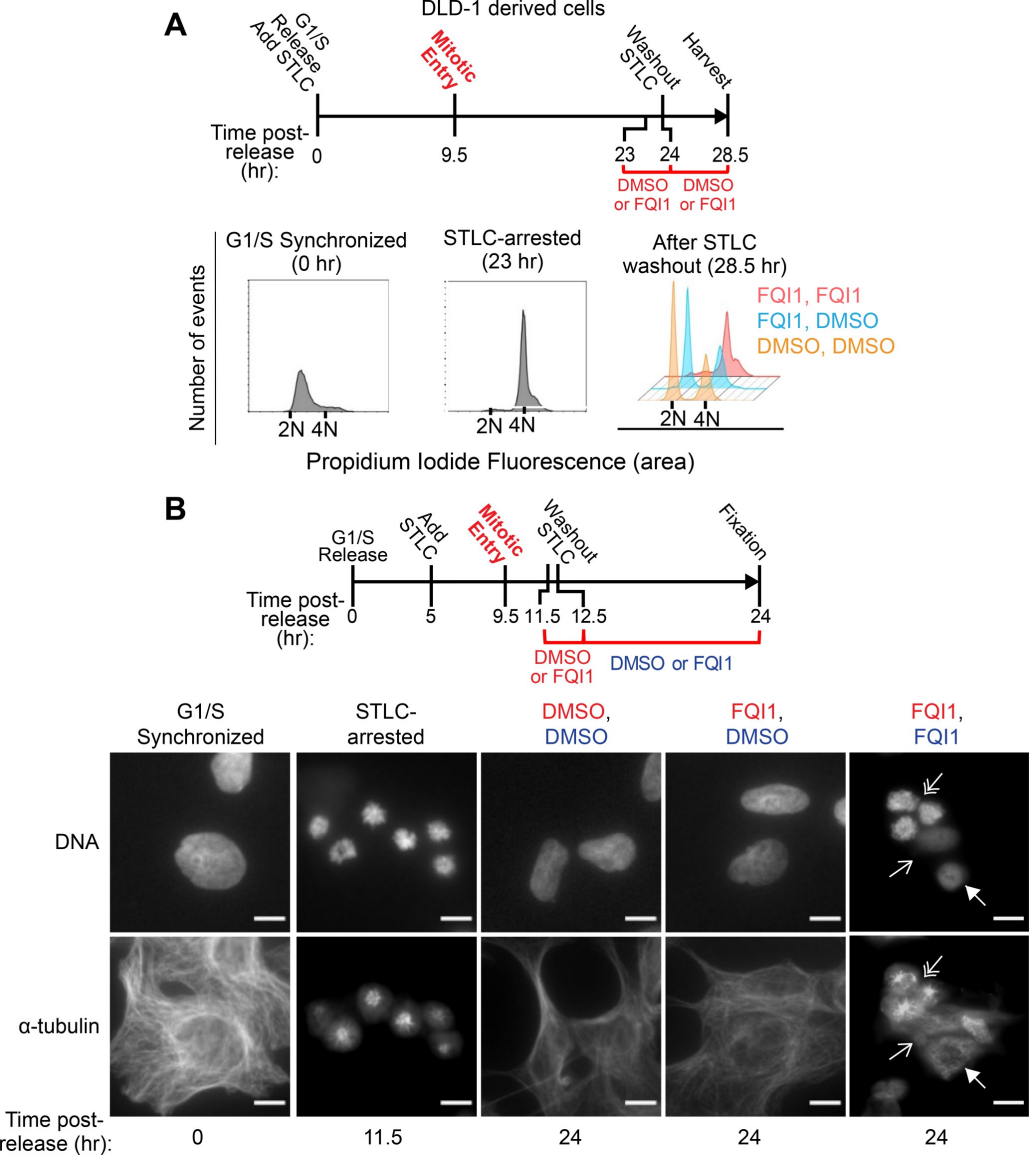
FQI1 addition in early mitosis is sufficient to induce rapid, reversible mitotic arrest. (A) Top: Schematic for treatments of synchronized DLD-1 derived cells following release from a single thymidine block. At the indicated time points, DNA content was profiled. At the final timepoints (28.5 hours): orange, control; blue, 4 μM FQI1-treated for 1 hour only; red, maintained in 4 μM FQI1. Representative of three experiments with 10,000 events analyzed for each treatment. (B) Left: Schematic for treatment of DLD-1 derived cells following release from a single thymidine block. Right: Representative immunofluorescent images of DNA and α-tubulin, harvested at the indicated times and treatment conditions. Cells mitotically arrested with STLC exhibited monopolar asters. Cells harvested at 24 h were maintained either in vehicle (DMSO) or 4 μM FQI1 for 12.5 h, or treated with 4 μM FQI1 for only 1 h, followed by vehicle for 11.5 h, as indicated by the color of the letters. Scale bars are 10 μm for all images. Cells maintained in FQI1 throughout are representative of a total of 56 cells from 8 images, demonstrating multiple mitotic-related defects, including arrest in mitosis (3 cells at double arrows) and multiple phenotypes of cells aberrantly exiting mitosis with condensed DNA (closed arrow) or diminished DNA signal and aberrant cytoskeletal structure and cell spreading (open arrow).

As a direct assessment of whether FQI1 addition to mitotic cells would block further progression, we employed the reversible mitotic inhibitor S-trityl-L-cysteine (STLC) [28]. STLC arrests cells early in mitosis with monopolar spindles. A derivative cell line of DLD-1 pseudodiploid colorectal cancer cells, in which STLC is well-tolerated [29], was synchronized by adding STLC following release from an initial G1/S arrest (Fig 2A). These mitotically arrested cells were exposed briefly to vehicle or 4 μM FQI1, followed by removal of STLC. A large fraction of control cells and cells exposed to FQI1 for only 1 hour then reentered G1, indicating substantial reversal from both STLC and FQI1 effects, despite lengthy incubation with STLC. In contrast, maintenance in FQI1 prevented cell division (Fig 2A). Analyzing a similar experiment by staining for α-tubulin and DNA confirmed the reversibility of FQI1-derived phenotypes, with cells returning to interphase following a 1-hour treatment in mitosis (Fig 2B: FQI1, DMSO). [Note that DLD-1 cells flatten out extensively in interphase, spreading until touching adjacent cells. Although mitotic cells appear to be profoundly smaller, they are simply rounded up; scale bars represent the same lengths in all images.] In contrast, continued presence of FQI1 prevented cells from appropriately dividing (Fig 2B: FQI1, FQI1), with a substantial fraction of cells remaining in a prometaphase-like state (double arrows), and others exhibiting multiple aberrant phenotypes (arrows). Parental DLD-1 cells similarly failed to undergo appropriate cell division when maintained in FQI1, resulting frequently in apoptosis (panel B in S1 Fig). These multiple aberrant phenotypes are consistent with previously reported FQI1-mediated mitotic defect-related phenotypes in other cell lines [5, 6].

If FQI1 disrupted mitosis due to transcriptional interference, it would necessitate that critical mitotic genes sensitive to FQI1 were being transcribed in mitosis. However, the limited transcription occurring in mitotically arrested cells is largely of genes involved in extracellular matrix and transcription processes [30]. Mitotic transcription important for progression through mitosis has been reported only for centromeres and *CCNB1* [31–33]. Unlike FQI1-mediated defects [6], inhibition of centromere transcription results in defects only later in mitosis, involving increased lagging chromosomes during anaphase [33]. Regarding *CCNB1*, although elevated cyclin B1 levels in asynchronously growing cells were initially suggested to cause FQI1-mediated mitotic arrest [5], further analysis using synchronized cells established that this was incorrect, with no alteration of cyclin B1 expression during FQI1-induced arrest [6]. Finally, RNA-seq analysis of synchronized HeLa cells harvested by mitotic shake-off, revealed no significant differences between FQI1-treated and control cells (Patrick Stoiber, personal communication). Thus, rapid and reversible mitotic arrest caused by FQI1, even when administered directly in mitotic cells, argues against these mitotic defects being caused by transcriptional dysregulation.

### FQI1 induces concentration-dependent defects in formation of the mitotic spindle

Previous mitotic phenotypes reported for FQI1 focused on chromosomal consequences, in particular with the persistence of condensed but unaligned chromosomes [5, 6]. Since stable attachment of microtubules to kinetochores is essential for achieving chromosomal alignment at metaphase [34, 35], we screened instead for spindle phenotypes generated by FQI1 treatment. Synchronized HeLa cells treated with 5 μM FQI1 upon release from a G1/S block notably resulted in mitotic cells with multiple α-tubulin foci (panel A in S2 Fig). Even at lower concentrations, this phenotype was relatively prominent, occurring in 3 and 18% of cells with 1.8 and 3.6 μM FQI1, respectively. To rule out effects of cell cycle synchronization or treatment during S and G2 in generating such multi-asters, spindle phenotypes were then quantified in asynchronous HeLa cells following 1-hour treatments with increasing concentrations of FQI1. Even at 0.5 and 1 μM FQI1, multi-asters and multipolar spindle formation were apparent in the mitotic cell population (panel C in S2 Fig). At 2.5 and 5 μM FQI1, with complete mitotic arrest prior to anaphase (panel B in S2 Fig), roughly half of mitotic cells displayed multi-asters/multipolar alignments (panel C in S2 Fig).

**Fig 3:**
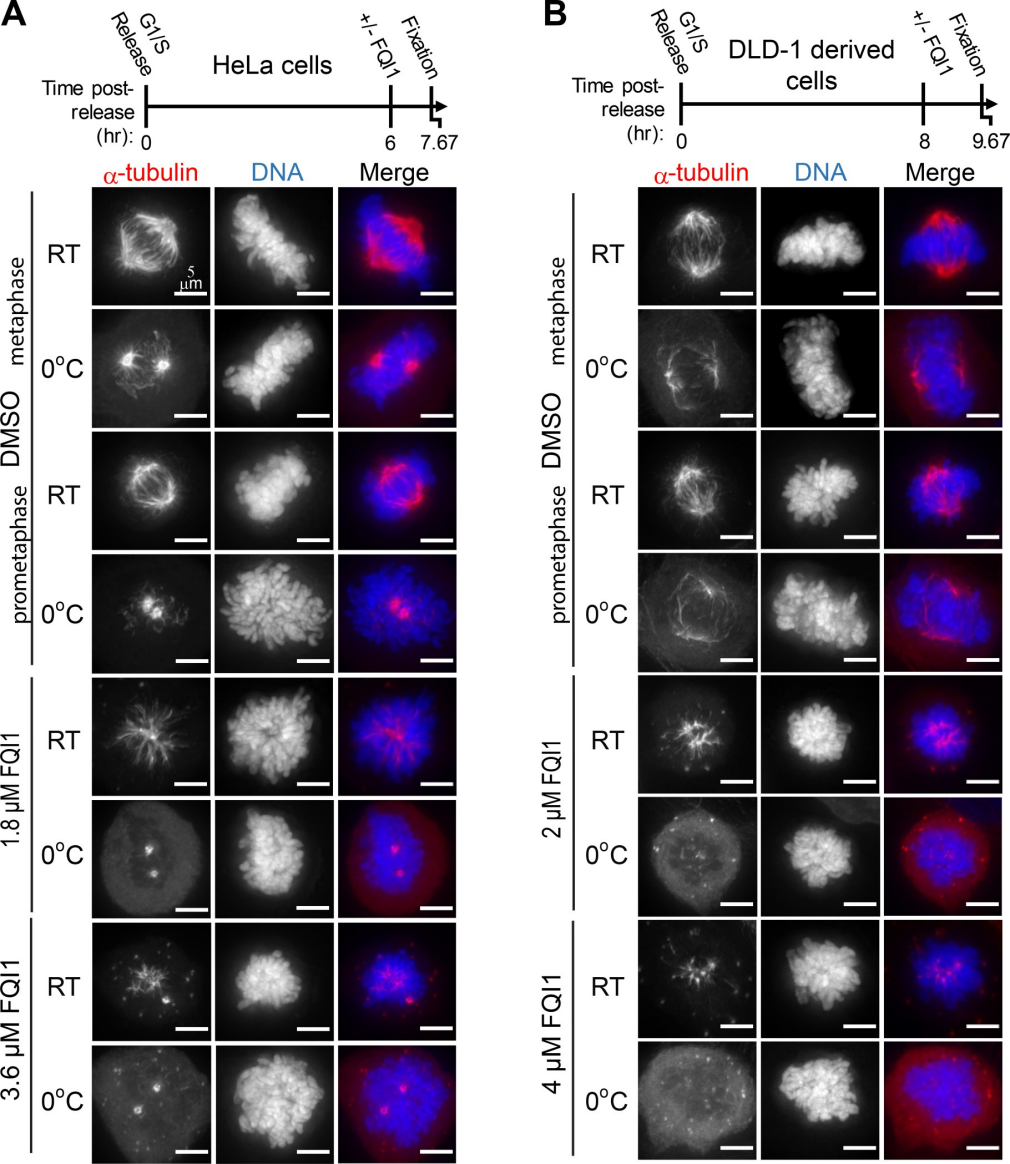
FQI1 induces spindle defects in a concentration-dependent manner and reduces stable MT-kinetochore attachments. (A) Top: Schematic of treatments of HeLa cells following release from a double thymidine block. Just prior to mitotic entry, cells were incubated with vehicle (DMSO), 1.8 μM FQI1, or 3.6 μM FQI1 for 100 minutes, and then fixed at ambient temperature (RT) or after ice water incubation (0°C). Images are representative from 3 independent biological experiments, with images totaling 21–27 cells per condition. Scale bars are 5 μm. (B) Top: Schematic for treatments of DLD-1 derived cells following release from a double thymidine block. Treatments just prior to mitotic entry included vehicle (DMSO), 2 μM FQI1, or 4 μM FQI1. Cells were analyzed as described in (A). Images are representative from 2–3 independent biological experiments, with images totaling 16–24 cells per condition.

Higher resolution analysis was performed in synchronized cells entering mitosis after brief treatments with two FQI1 concentrations. Immediately prior to fixation, cells were incubated at either ambient or cold temperature to assess microtubule stability. Whereas most microtubules depolymerize at cold temperatures, microtubules stably attached to kinetochores are cold-resistant [36–38]. When fixed at ambient temperature, both HeLa and DLD-1 derived cells demonstrated a concentration-dependent disruption in spindle formation following brief FQI1 exposures (Fig 3, “RT” samples). Additionally in these samples, an excess of α-tubulin foci was evident, particularly at the higher FQI1 concentrations, consistent with the previous lower resolution screen. Furthermore, whereas incubation at 0°C diminished the numbers of microtubule fibers in control cells, leaving only kinetochore-attached microtubules, cold treatment of cells incubated with FQI1 essentially left no remaining microtubules. This disruption of spindles, associated with the lack of stable microtubule-kinetochore attachments, is likely the primary cause of the FQI1-mediated mitotic arrest.

Cancer cells frequently exhibit supernumerary centrosomes [39, 40]. To avoid these confounding issues, and to determine whether microtubule defects mediated by FQIs are more generally observed, we investigated consequences of FQI1 in the near-diploid and mitotically normal, immortalized retinal pigmented epithelial (RPE) cells [41].

**Fig 4:**
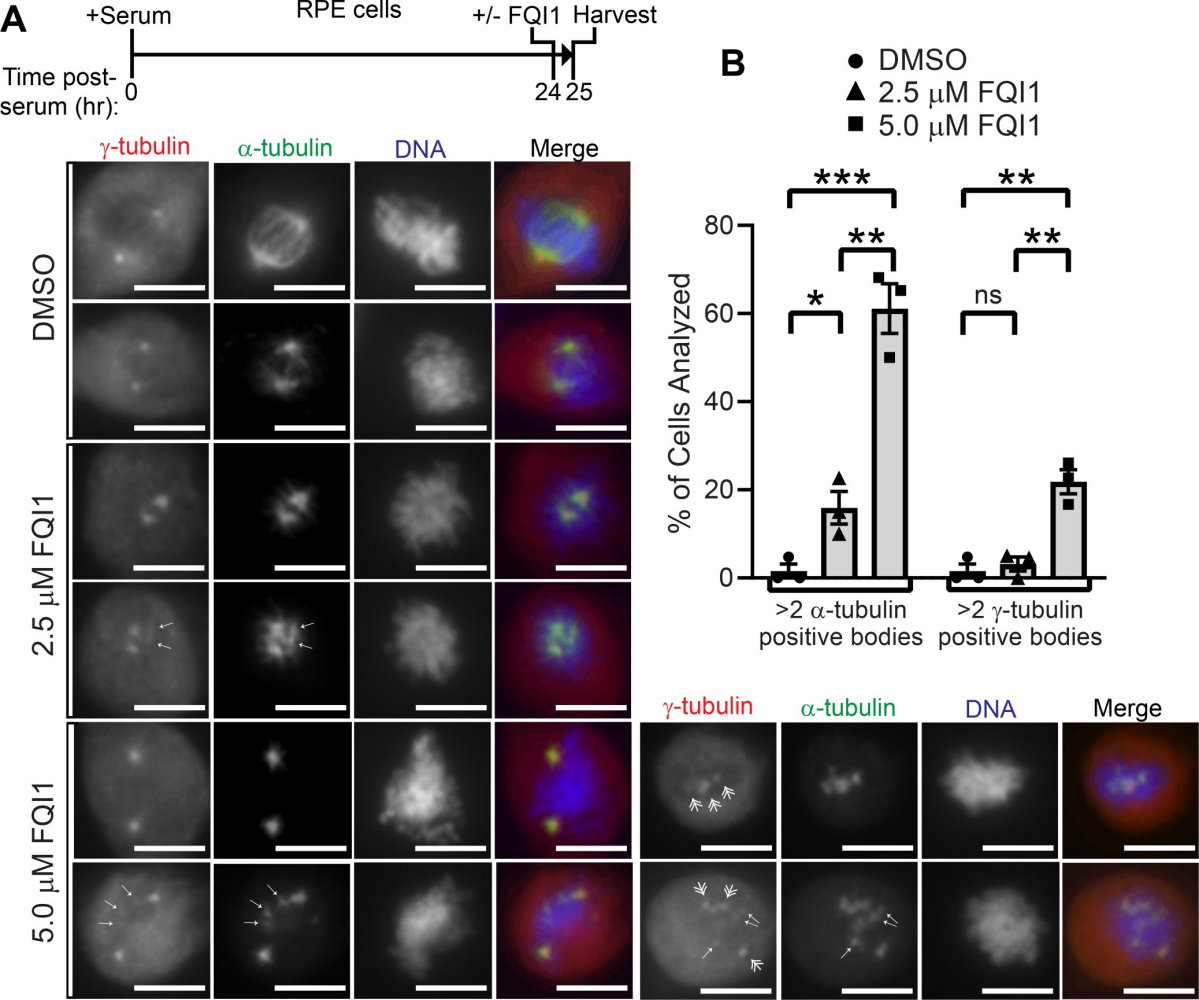
Dose-dependent induction by FQI1 of extra α-tubulin- and γ-tubulin-positive structures. (A) Top: Schematic of treatments of synchronized RPE cells in quiescence by serum starvation. Bottom: Representative images of cells stained for γ-tubulin, α-tubulin and DNA from a total of at least 60 cells over three independent biological experiments for each treatment. Scale bars are 8 μm. (B) Quantification of the percentage of cells containing more than 2 α-tubulin positive bodies or more than 2 more prominently staining γ-tubulin positive bodies under each condition. Cells in which <2 γ-tubulin spots were identified were excluded, as the centrosomes likely had not separated, preventing accurate quantification. Shown are averages of three biological replicates with error bars representing SEM. P values were calculated using an unpaired 2-tailed t-test. For >2 α-tubulin positive bodies: *p = 0.024; **p = 0.0026; ***p<0.001. For >2 γ-tubulin positive bodies: **p = 0.0031 (0 vs 5 μM FQI1); **p = 0.0043 (2.5 vs 5 μM FQI1); ns = not significant.

The relationship between multi-asters and γ-tubulin, a key component in nucleating microtubules, was analyzed by treating synchronized RPE cells for 1 hour with FQI1 as they entered mitosis, followed by staining for both α-tubulin and γ-tubulin (Fig 4A). Controls verified minimal, if any, spectral overlap in the fluorescence signals detecting α- versus γ-tubulin (S3 Fig). In vehicle-treated cells, γ-tubulin overlapped with α-tubulin at the spindle poles, as expected. Similar to the cancer cell lines, incubation with FQI1 led to diminished spindle formation and a concentration-dependent increase in cells containing extra α-tubulin foci. In general, each extra α-tubulin feature coincided with a much fainter extra γ-tubulin feature (arrows, Fig 4A). Extra asters appeared in a significant fraction of cells (16%) at 2.5 μM FQI1, and in the majority of cells (61%) at 5 μM FQI1 (Fig 4B). Various mechanisms could achieve such multi-asters, including lack of resolution of acentrosomal γ-tubulin-dependent microtubule nucleation [42, 43]. At the higher FQI1 concentration, one, or occasionally more, additional prominent γ-tubulin structures (double arrows, Fig 4A) were also present in a significant percentage of the cells (21%) (Fig 4B). These additional bodies may likely represent pericentriolar material fragmentation, which can be induced rapidly after onset of mitotic delays [44], including in RPE cells mitotically delayed for just one hour [45].

Overall, the mitotic consequences of FQI1 across multiple cell types are similar: condensed but unaligned chromosomes [6], with concentration-dependent spindle abnormalities. For cancer cells lacking robust mitotic checkpoint control, the consequence of such abnormalities frequently include aberrant cell division leading to death or senescence, as has been documented for FQI1 [6].

### Mitotic arrest potency of FQIs tracks with potency in inhibiting LSF

**Fig 5:**
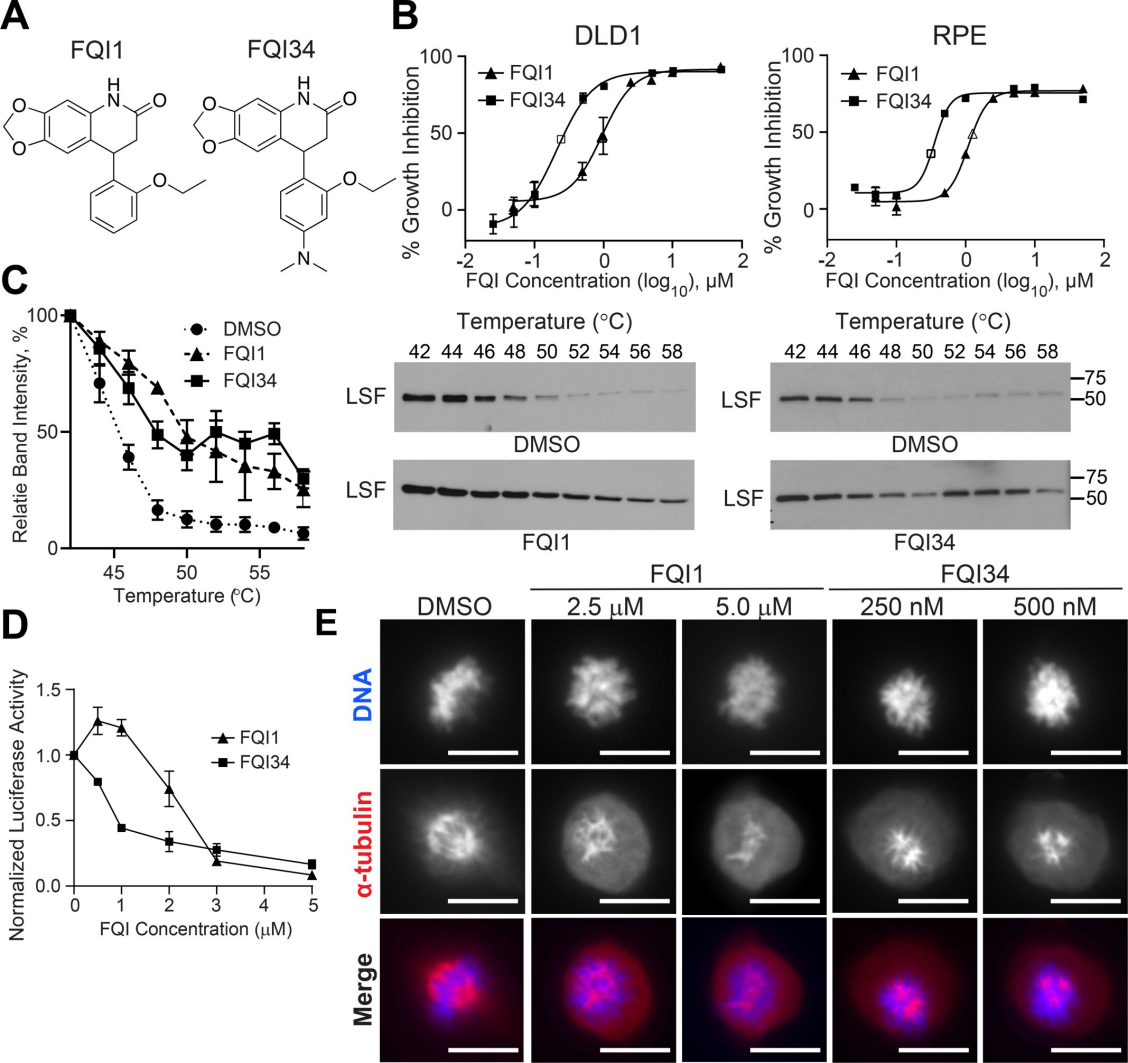
FQI34, a more potent LSF inhibitor, disrupts mitosis with spindle defects at 10-fold lower concentrations than FQI1. (A) Comparison of structures of FQI1 and FQI34. (B) MTS assays measuring the number of viable cells upon treatment of parental DLD-1 cells (left) and RPE cells (right) with increasing concentrations of FQI1 versus FQI34. Calculated GI50’s are: DLD-1 FQI1, 0.95 μM; DLD-1 FQI34, 0.24 μM; RPE FQI1, 1.25 μM; RPE FQI34, 0.32 μM. Data indicate averages +/- SEM from 6 replicates. (C) CETSAs showing increase in LSF thermal stability in presence of FQI1 versus FQI34. Left: Quantitation of remaining soluble LSF at indicated temperature, with averages +/- SEM from 3 independent experiments. Representative immunoblots of the primary data from FQI1 (middle) and FQI34 (right) CETSAs. (D) Inhibition of LSF-dependent reporter assays in cells treated with increasing concentrations of FQI1 versus FQI34. The reporter plasmid, with an LSF-dependent promoter driving luciferase expression, is described in S4 Fig (panel B). Data are averages +/- SEM from 3 independent experiments. (B-D) Detailed protocols are described in S1 File. (E) Representative images of RPE-hTERT-Flp-In cells synchronized and treated as in the schematic shown in Fig 4A, using the indicated concentrations of either FQI1 or FQI34. Cells were stained for α-tubulin and DNA.

As an initial test of whether the induction of mitotic arrest by FQIs might be related to inhibition of LSF, we tested whether this phenotype tracks with the efficacy of the inhibitor against LSF. The more potent LSF inhibitor FQI34 [46] (Fig 5A) exhibited a ~4-fold lower GI_50_ concentration than FQI1 in both DLD-1 parental cells and RPE cells (Fig 5B). Both compounds enhanced the thermal stability of LSF, as shown by cell thermal shift assays (CETSAs, Fig 5C), supporting that they interact directly with LSF. Finally, both compounds inhibited the transcriptional activity of LSF, with FQI34 being approximately 3-fold more potent in reducing the activation of an LSF-dependent reporter than was FQI1 (Fig 5D). Based on these relative potencies, effects of the compounds on RPE cell cycle progression were analyzed by microscopy and visualization of both α-tubulin and DNA. Both compounds caused defects in mitotic progression of RPE cells, with aberrant spindles, multi-asters, and condensed but unaligned chromosomes, with FQI34 being similarly effective at even 10-fold lower concentrations than those of FQI1 (Fig 5E). These results were consistent with mitotic defects being related to LSF inhibition.

### LSF facilitates tubulin polymerization *in vitro*

**Fig 6:**
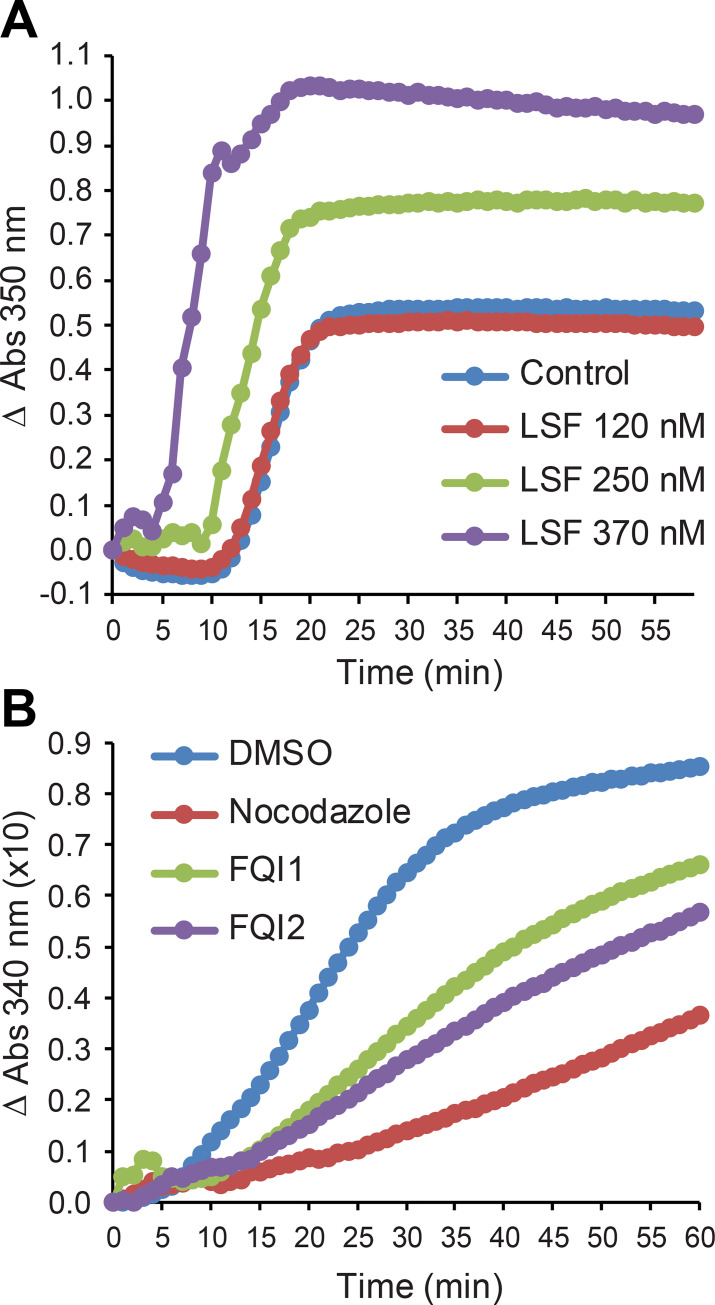
LSF facilitates tubulin polymerization *in vitro*. (A) The tubulin polymerization assay was performed under standard conditions (Millipore kit) in the presence of 52 μM tubulin and 0 nM (blue), 120 nM (red), 250 nM (green) and 370 nM (purple) LSF. (B) The tubulin polymerization assay was performed under standard conditions (Cytoskeleton kit) in the presence of vehicle (DMSO, blue), 5 μM FQI1 (green), or 5 μM FQI2 (purple). Nocodazole (5 μM, red) is a positive control for polymerization inhibition.

Microtubule dynamics are modulated by interactions between tubulins and other proteins, including microtubule motor proteins and non-motor microtubule-associated proteins [47]. Since LSF has been identified as a microtubule-associated protein [12], we tested whether it might be capable of modulating microtubule dynamics. The standard *in vitro* tubulin polymerization assay involves monitoring by optical density the formation of polymerized microtubules over time [48]. Highly purified cellular tubulin preparations containing MAPs are generally used, to permit efficient polymerization kinetics, since purified recombinant α- and β-tubulins do not polymerize efficiently. Thus, prior to polymerization, limiting amounts of LSF were added to tubulin at molar ratios ranging from 1:140–430 LSF:tubulins. The slope of the linear part of the absorbance curve indicates the rate of polymerization. Starting at a ratio of added LSF:tubulins of 1:210, the rate of tubulin polymerization increased, by 1.4-fold at 250 nM LSF and 1.6-fold at 370 nM LSF. Thus, LSF significantly enhanced the initial rate of polymerization (Fig 6A).

Since LSF is detectable within the initial tubulin preparation [12], and FQI1 interrupted the LSF-tubulin interaction in vivo [12], we tested whether addition of FQIs to the tubulin polymerization assay even in the absence of added LSF would inhibit polymerization. Indeed, both FQI1 and FQI2 [1] inhibited tubulin polymerization *in vitro* at concentrations known to fully inhibit LSF (Fig 6B). Although this result is consistent with FQIs reversing the ability of LSF to enhance tubulin polymerization, it is certainly not definitive in that regard. It would also be consistent with several other scenarios, including inhibition of tubulin polymerization by directly interacting with microtubules.

### FQI1 inhibits associations between biotinylated LSF and microtubule-associated proteins

Critical to understanding the mechanism causing these FQI1-mediated mitotic phenotypes remains identification of the direct FQI1 target(s). Multiple indications strongly support that FQIs generally quite specifically target LSF [1, 4, 12, 20], including but not limited to: 1) the IC_50_ for LSF inhibition and the GI_50_ for cell growth inhibition of FQIs correlate linearly, with an approximate slope of one [4], and 2) FQI1 and siRNA specifically targeting LSF cause similar mitotic delays with condensed but unaligned chromosomes [6]. Our results argue against transcriptional dysregulation as the cause of the FQI1-induced defects, despite LSF being a transcription factor. However, since FQI1s can also disrupt LSF-protein interactions, and other transcription factors are documented to play non-transcriptional roles in mitotic structural components [13], we hypothesized that spindle defects result from FQI1 inhibition of LSF-protein interactions required to generate and/or stabilize mitotic spindles. Notably, in addition to directly binding α-tubulin, LSF binds SET8 methyltransferase, enhancing methylation of α-tubulin at K311 [12]. Although the consequence of this particular methylation is not yet known, tubulin post-translational modifications impact multiple aspects of microtubule dynamics, including promotion of stable kinetochore-microtubule attachment [49]. Thus, LSF could influence microtubule dynamics either by affecting microtubule stability directly or by synergizing with other microtubule-associated proteins, such as SET8 [9, 12, 50]. In our previous study, FQI1 treatment in asynchronous HEK293 cells partially diminished the level of K311 methylation on α-tubulin, although the effect was subtle [12]. The rate of demethylation would depend on the presence of a demethylase, which has not been identified. Therefore, rather than focusing on SET8 as the prime target of LSF in affecting microtubule stability, we sought to identify a list of candidate interacting proteins that could rapidly impact spindle formation, if interrupted.

To screen for associated proteins in mitosis whose interactions with LSF are FQI1-sensitive, we used as bait endogenously biotinylated LSF (BioLSF, panel A in S4 Fig) that was inducibly expressed in the presence of doxycycline. This BioLSF fusion protein retains LSF activity, as measured by the ability to activate expression from a LSF-dependent reporter construct (panel B in S4 Fig). DLD-1 derived cells expressing BioLSF were incubated for one hour in mitosis with either 4 μM FQI1 or vehicle, and were treated with formaldehyde prior to harvest in order to crosslink and maintain weak or transient protein-protein interactions. Cell lysates were incubated with streptavidin beads in order to capture BioLSF and its associated proteins. Given the high affinity biotin-streptavidin interactions, stringent washing conditions could be used to limit nonspecifically bound proteins. As additional controls to eliminate background proteins from consideration during data analysis, parallel mitotic cell lysates were prepared from parental DLD-1 cells that lack the integrated BioLSF cDNA, and from uninduced DLD-1 derived cells, in which only leaky, minimal BioLSF expression is detectable (panels C and D in S4 Fig). Proteins eluted from the streptavidin beads, including two biological replicates of the entire set of lysates, were identified by mass spectrometry. Notably, this methodology could identify not only proteins that directly interact with BioLSF, but also proteins in complexes with direct BioLSF partner proteins.

For the analysis, as an initial non-stringent cut, proteins bound to the streptavidin beads from parental DLD-1 lysates, as well as proteins whose binding to the beads did not increase upon BioLSF induction in DLD-1 derived cells, were removed. Of the remaining proteins (S1 Table, filtered hits), gene ontology analysis indicated statistically overrepresented biological functions including cell-cell adhesion and cell division, as well as protein translation and mRNA splicing (S2 Table). Although the translation and splicing machinery were initially unexpected for mitotic lysates, both translation and splicing components have been reported to associate with mitotic spindles, and in individual cases to be required for proper assembly of the spindle [13, 51, 52]. Thus, these categories were consistent with the ability of LSF also to directly bind α-tubulin [12]. In addition, transcription factors do interact with RNA processing machinery, and in the case of one LSF-regulated gene, thymidylate synthase, high-affinity and regulatory binding sites are located near intron-exon boundaries [53], consistent with roles in splicing regulation.

When analyzed for sensitivity to FQI1, and therefore potentially involved in the FQI1-mediated mitotic phenotypes, BioLSF associations with roughly one third of the proteins were decreased at least 2-fold after treatment with FQI1 (S1 Table, FQI1 decreases). These FQI1-sensitive potential partner proteins were statistically overrepresented in cell-cell adhesion, cell division, and mRNA splicing categories, but not translation machinery (S3 Table). Only 48 proteins exhibited enhanced association (2-fold or greater) with BioLSF in the presence of FQI1 (S1 Table, FQI1 increases), and overrepresentation in a single functional category (S4 Table). Overall, these results suggest there is specificity of BioLSF associating proteins that are sensitive to disruption by FQI1.

Of the FQI-sensitive proteins, thirteen have well-documented roles in mitotic spindle and/or mitotic centrosomal assembly and function (Table 1). The signal intensities of all of these proteins were substantially increased in cell lysates in which BioLSF was inducibly expressed (+Dox), and were all decreased greater than 2-fold upon treatment with FQI1 (+FQI1). In addition, multiple functional protein-protein interactions among this set have already been established (Table 1). Thus, these BioLSF associated proteins from the mass spectrometry screen are candidate mediators, along with LSF, of the FQI1-mediated mitotic defects. To date, nothing is known regarding which of these LSF-protein interactions may be required in normal mitotic progression to establish and maintain spindle pole integrity and spindle formation, leading to stable microtubule-kinetochore attachment and progression through mitosis. Here, we propose possible mechanisms, which are not mutually exclusive.

**Table 1:** FQI1-sensitive BioLSF-interacting proteins involved in mitotic spindle assembly, positioning, and dynamics or centrosome integrity, positioning, and function.

Protein	Gene Name	Fold increase + Dox^a^	Fold decrease + FQI1^a^	Interactions with LSF partners	Mitotic function(s) related to FQI1 phenotypes^c^
Aurora kinase A	AURKA	>13	2.5	TPR [71]	Regulates centrosomes Regulates spindle establishment
Cyclin-dependent kinase 1	CDK1	≥31	2.1		Regulates mitotic onset
					Regulatesspindle dynamics
Tubulin beta-6	TUBB6	57	6.1		Minor beta-tubulin
					Reduces MT stability
					Normal mitotic progression
Cytoskeleton-associated protein 5	CKAP5	≥50	2.1		Spindle pole maintenance
					Processive + tip tracking
Kinesin family member 2C	KIF2C	≥14	3.4		Depolymerizes spindle MTs at + ends
					Corrects improper MT-kinetochore attachments
Ran GTPase-activating protein 1	RANGAP1	45	4.1	RANBP1 [84]	Bound to kinetochores complexed with RANBP2
					Spindle localization
Ran binding protein 1	RANBP1	≥22	6.3	RANGAP1 [84]	Facilitates GTP hydrolysis of RANGTP
					Bound to centrosomes
					Normal spindle assembly
Cytoplasmic dynein 1 heavy chain 1	DYNC1H1	≥81	3.0	TPR [85]	Mitotic spindle pole assembly and maintenance
				CTNNB1 [86]	
Cytoskeleton-associated protein 2	CKAP2	16	2.8		Enhances MT nucleation
					Bundles MTs
					Centrosome integrity
Nucleoprotein TPR	TPR	≥14	5.1	AURKA [71]	Centrosome maintenance, AURKA activation Prometaphase kinetochore localization
				DYNC1H1 [85]	
RuvB-like AAA ATPase 1	RUVBL1	≥18	2.7	CTNNB1 [87]	Assembly of γ-TURC complexes
					Centrosome and spindle localization
Beta-catenin	CTNNB1^b^	45	3.5	RUVBL1 [87]	Centrosomes/spindle pole localization
				DYNC1H1 [86]	Centrosome separation in early mitosis
Kinesin family member 23	KIF23	≥9	≥9		Cytokinesis, as centralspindlin component
					Bundles antiparallel MTs

^a^Values based on signal intensities. Where divisor was 0, minimal ratios were calculated.

^b^Previously reported direct interaction with LSF [76]

^c^MT = microtubule

First, it is important to note that the function of LSF, including its ability to interact with specific proteins, is likely to be influenced by its modification state, as is the case for many mitotic proteins. The BioLSF interactions with AURKA and CDK1 suggest that LSF is a substrate of these two critical mitotic kinases: AURKA regulates many aspects of spindle pole assembly and function [54, 55], and CDK1 is not only the key regulator of mitotic entry, but also phosphorylates proteins involved in numerous aspects of mitotic control, particularly leading up to anaphase [56, 57]. With regards to CDK1 in particular, we previously demonstrated that LSF is phosphorylated at multiple SP or TP motifs, and that LSF activity is thereby modulated by the phosphorylation-specific prolyl isomerase Pin1 [58]. Given that Pin1 is expressed at highest levels in mitosis, these findings are consistent with an ability of CDK1 to modify LSF functionality.

With regards to generation and maintenance of a stable bipolar spindle, the interaction of BioLSF with TUBB6, a minor β-tubulin, is particularly intriguing. This β-tubulin is highly unusual in that it inhibits both tubulin acetylation and overstabilization of microtubules [59]. Furthermore, overexpression of TUBB6 leads to disrupted spindles and fragmented microtubules [60]. We therefore propose that association of LSF with TUBB6 normally moderates the ability of this atypical tubulin to destabilize the spindle.

Regarding the FQI1-mediated reduction of stable microtubule-kinetochore fibers, interaction with LSF may normally facilitate the appropriate localization or activity of the following FQI1-sensitive BioLSF-binding proteins: CKAP5, which protects microtubule plus ends, facilitates microtubule polymerization, and stabilizes microtubule-kinetochore attachment [61–63], KIF2C (also named MCAK), which depolymerizes microtubules at the plus ends, specifically correcting inappropriate microtubule-kinetochore attachments [64], and/or RANGAP1, which localizes to kinetochores through association with RANBP2, thereby stabilizing microtubules that are appropriately attached to kinetochores [65].

Although the most obvious phenotype of FQI1 treatment is to disrupt spindle formation, these consequences could also be accomplished less directly by interfering with the microtubule nucleation and/or bundling that are required to establish the bipolar spindle. Indeed, a number of proteins that impact centrosome integrity and centrosome-mediated microtubule nucleation were identified as FQI-sensitive BioLSF-associated proteins. The centrosome consists of a complex structure in which the machinery for nucleation and attachment of the microtubule minus ends is embedded in the pericentriolar material. Knockdown of the following proteins causes disruption of centrosome integrity, leading to multiple spindle poles: RANBP1, which controls appropriate recruitment of proteins in mitosis by regulating the RANGTP gradients [66–68], dynein light chain subunits [69], CKAP2 [70], AURKA, and TPR, which promotes AURKA activation [71]. The potential association of LSF and CKAP2 is of particular interest, since CKAP2 at substoichiometric levels dramatically enhances microtubule nucleation and bundling [72], which is similar to our data regarding LSF (Fig 6), suggesting the possibility that CKAP2 and LSF work in concert to promote spindle formation. Finally, RUVBL1, which localizes to both centrosomes and spindles [73], functions as a chaperone co-activator for formation of quaternary protein complexes [74]. In particular, RUVBL1 it is essential for the assembly of active γ-TURC complexes, at which microtubules are nucleated [75]. Thus, LSF interaction with RUVBL1 suggests that LSF might also be incorporated into such complexes in order to facilitate microtubule nucleation.

Bipolarity of the spindle also requires β-catenin, which has previously been demonstrated to associate with LSF [76]. Beta-catenin localizes to the centrosomes throughout the cell cycle, and in response to phosphorylation by Nek2 in mitosis, it triggers centrosome separation [77, 78]. Either knockdown of β-catenin or expression of a mutant incapable of phosphorylation by NEK2 results in monopolar spindles, which is similar to the phenotype from FQI1 addition. Thus, binding of LSF to β-catenin might either block β-catenin phosphorylation by NEK2 or prevent the subsequent triggering event, as yet unknown, that leads to centrosome separation.

Other mechanisms for the generation of multiple spindle poles are lack of focusing of microtubules that are not nucleated and attached at centrosomes, or inappropriate detachment of microtubules from centrosomes. The alternate route of microtubule nucleation occurs around kinetochores, where newly nucleated microtubules are captured. During active centrosome-mediated nucleation, this alternative mode is downregulated by the binding of a RANGAP1/RANBP2 complex to kinetochores [65, 79, 80], although a mechanism for how LSF association with RANGAP1 would normally prevent inappropriate nucleation and/or enhance capture is not obvious. Two other FQI1-sensitive interactors, dynein [81] and CKAP2 [72] normally promote focusing of microtubules at the spindle poles, in order to maintain bipolarity. Considering the similar functions of CKAP2 and LSF discussed above, LSF could also normally facilitate CKAP2-mediated microtubule focusing, to prevent multi-aster formation. Finally, overexpression of two proteins whose interactions with BioLSF are FQI1-sensitive induce centrosome detachment: KIF2C [82] and TUBB6 [83]. Under normal mitosis, binding of LSF might normally prevent block these aberrant activities.

Overall, the BioLSF mitotic interactome analysis remains consistent with the model that LSF is a target of FQIs in mitosis, and that spindle defects could result from disruption of key LSF-protein interactions that facilitate normal spindle formation and mitotic progression. Given the many possible models, additional detailed investigations are required to identify which LSF-protein interactions regulate normal mitotic progression.

## Conclusions

The previously identified FQI1 target, LSF, promotes cancer growth and metastasis in multiple cancer types [1–3, 76, 88, 89], in part through enhancing expression of proliferation and invasion-promoting genes. However, the mitotic defects induced by both FQI1 and LSF siRNA suggested that LSF also directly regulates mitotic progression [6]. The ability of FQIs to diminish interactions between BioLSF and microtubule- and centrosome-associated proteins does support the hypothesis that such interactions between LSF and partner proteins normally promote spindle formation and/or maintenance. Further studies are required to prove this model and to pinpoint which FQI1-sensitive LSF-protein interactions might be key to establishment of a normal spindle and alignment of chromosomes in metaphase.

Several cancer chemotherapeutics in use do target microtubule dynamics, although the current consensus is that their therapeutic potential derives from effects on microtubule functions in interphase. Our recent study demonstrates that FQIs do interfere with normal microtubule structure and modifications in interphase cells [90]. Whether or not disruption of either the mitotic spindle or interphase microtubules contributes significantly to the anti-tumor activity of FQIs remains to be determined. However, the distinct molecular mechanisms underlying microtubule disruptions proposed here may explain how FQIs inhibit tumor growth in animal studies without detectable toxicity [1, 5, 91], unlike the serious side effects common for direct microtubule-binding inhibitors [10, 92, 93].

## Supporting information

S1 Fig Rapid induction by FQI1 of mitotic arrest in multiple cell lines. (A) Left: Schematic for treatment of HeLa cells following release from a double thymidine block. Cells treated with the indicated concentrations of FQI1 30 minutes prior to approximate mitotic entry, and were analyzed at the indicated times for cellular DNA content by propidium iodide staining and flow cytometry. Right: Profiles of cellular DNA content for G1/S synchronized cells and the cells approximately 2.5 hr after mitotic entry, incubated with the indicated treatments. Results are representative of three experiments with 10,000 events analyzed for each treatment. (B) Top: Schematic of treatments of synchronized DLD-1 parental cells following release from a single thymidine block. Bottom: Immunofluorescence analysis of DNA and α-tubulin, as indicated. “G1/S” indicates cells arrested at the thymidine block. “STLC-arrested” indicates cells arrested in mitosis with monopolar asters. Remaining images show cells harvested after treatment with either vehicle (DMSO) or 4 μM FQI1 during the time periods indicated by the color of the letters. Cells maintained in FQI1 throughout (FQI1, FQI1) are representative of a total of 243 cells from 18 images across two biological replicates, demonstrating multiple mitotic-related defects, including prometaphase-like cells and apoptotic cells (various types of arrows indicating different cells). Scale bars are 10 μm.
(TIF)

S2 Fig Induction by FQI1 of multi-aster formation and concentration-dependent spindle defects in HeLa cells. (A) Immunofluorescence of DNA and α-tubulin, as indicated, in mitotic HeLa cells. Cells were synchronized at the G1/S border by a double thymidine block and released in presence of 5 μM FQI1 or vehicle (DMSO). Fixed cells were stained with α-tubulin antibody and DAPI (DNA stain). Scale bars are 20 μm. Data are representative of 2 independent biological experiments. (B-C) An asynchronous population of HeLa cells (from the Shah laboratory) were treated with increasing doses of FQI1 for 1 h. Cells were analyzed by immunofluorescence for α-tubulin, γ-tubulin, and DNA. The mitotic index was similar amongst all the treatment groups, varying from 3–6%. (B) Each treatment group was analyzed for the percentage of mitotic cells that were pre- versus post-anaphase. (C) Mitotic spindles were classified for all mitotic cells: normal, bipolar cells, early prometaphase cells (also including early mitotic cells in which the centrosome were not significantly separated), and multipolar/multiaster cells. In the 2.5 μM FQI1 and the 5 μM FQI1 samples, there was also a general loss of α-tubulin staining.
(TIF)

S3 Fig Controls indicating lack of overlap in fluorescence channels detecting α-tubulin versus γ-tubulin. Immunofluorescence images of RPE cells treated as in Fig 4A, but stained for DNA and only one primary antibody, against either γ-tubulin (top) or α-tubulin (bottom), plus its respective secondary antibody. These images are representative from a total of 43–45 imaged cells across three independent biological experiments. Scale bars are 8 μm. These data verify the extremely minimal, if any, fluorescent emission bleed-through between the channels detecting the Alexa Fluor 546 and Cy5 fluorophores.
(TIF)

S4 Fig Structure and characterization of BioLSF. (A) Structure of the BioLSF fusion protein. Amino acid numbers are indicated. Bio: Biotinylated domain from BCCP; TAD: transcriptional activation domain of LSF; DBD: DNA binding domain of LSF; Oligomerization: a region encompassing both the LSF dimerization and tetramerization functions. (B) Dual-luciferase reporter assay to measure LSF transcriptional activity. Top: Schematic of the LSF-dependent firefly luciferase reporter construct. Bottom: Reporter activity of transfection of a BioLSF-expressing construct (BioLSF) compared to an empty vector control (-). Relative activity indicates the levels of LSF-regulated firefly luciferase activity normalized to that of the control Renilla luciferase activity. Firefly luciferase activity was increased 15-fold upon induction of BioLSF expression. Data points indicate averages of technical replicates from three independent biological experiments. Bars represent means ± SEM. Unpaired t-test, *p = 0.022. (C) Top: Streptavidin blot showing robust induction of biotinylated BioLSF upon doxycycline treatment for 24 and 48 hours of DLD-1 derived cells. Bottom: Blot for β-actin, as a loading control. Molecular weight markers are in kDa. Representative of at least three experiments. (D) Top: Blot using LSF antibody showing expression of BioLSF and LSF in DLD-1 derived lysates from uninduced and induced cells treated with doxycycline for 3.25 hours. Note the significant induction, equivalent to levels of endogenous LSF by 3.25 hours after induction. Also, note that in the tetracycline-free media, there remains some low level expression of BioLSF. Bottom: Blot for α-tubulin, as a loading control.
(TIF)

S1 Table Complete list of mitotic BioLSF-interacting proteins (non-stringent cut). (XLSX)

S2 Table Gene Ontology analysis of complete list of mitotic BioLSF-interacting proteins. (PDF)

S3 Table Gene Ontology analysis of mitotic BioLSF-interacting proteins reduced by FQI1. (PDF)

S4 Table Gene Ontology analysis of mitotic BioLSF-interacting proteins enhanced by FQI1. (PDF)

S1 File Preparation and characterization of reagents: FQIs, DLD-1 derived cell line, and BioLSF. (PDF)

S1 Raw images (PDF)
