# Supplementary figures and images for: Factor quinolinone inhibitors disrupt spindles and multiple LSF (TFCP2)-protein interactions in mitosis, including with microtubule-associated proteins
Source: PLoS One. 2022 Jun 15;17(6):e0268857. doi: 10.1371/journal.pone.0268857 (PMC9200292; doi:10.1371/journal.pone.0268857)

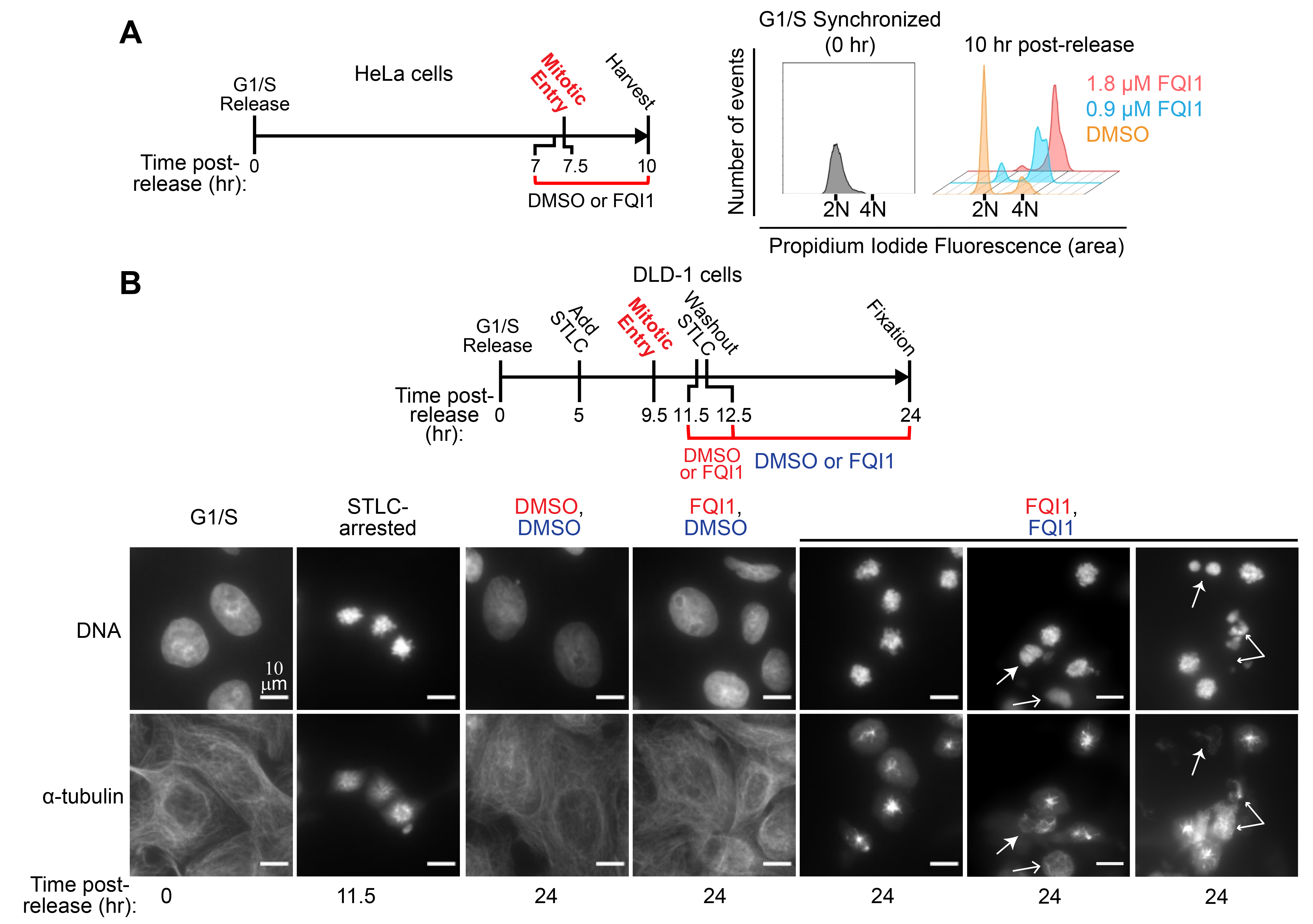

Supplement: S1 Fig — (A) Left: Schematic for treatment of HeLa cells following release from a double thymidine block. Cells treated with the indicated concentrations of FQI1 30 minutes prior to approximate mitotic entry, and were analyzed at the indicated times for cellular DNA content by propidium iodide staining and flow cytometry. Right: Profiles of cellular DNA content for G1/S synchronized cells and the cells approximately 2.5 hr after mitotic entry, incubated with the indicated treatments. Results are representative of three experiments with 10,000 events analyzed for each treatment. (B) Top: Schematic of treatments of synchronized DLD-1 parental cells following release from a single thymidine block. Bottom: Immunofluorescence analysis of DNA and α-tubulin, as indicated. “G1/S” indicates cells arrested at the thymidine block. “STLC-arrested” indicates cells arrested in mitosis with monopolar asters. Remaining images show cells harvested after treatment with either vehicle (DMSO) or 4 μM FQI1 during the time periods indicated by the color of the letters. Cells maintained in FQI1 throughout (FQI1, FQI1) are representative of a total of 243 cells from 18 images across two biological replicates, demonstrating multiple mitotic-related defects, including prometaphase-like cells and apoptotic cells (various types of arrows indicating different cells). Scale bars are 10 μm. (TIF) [file pone.0268857.s001.tif]

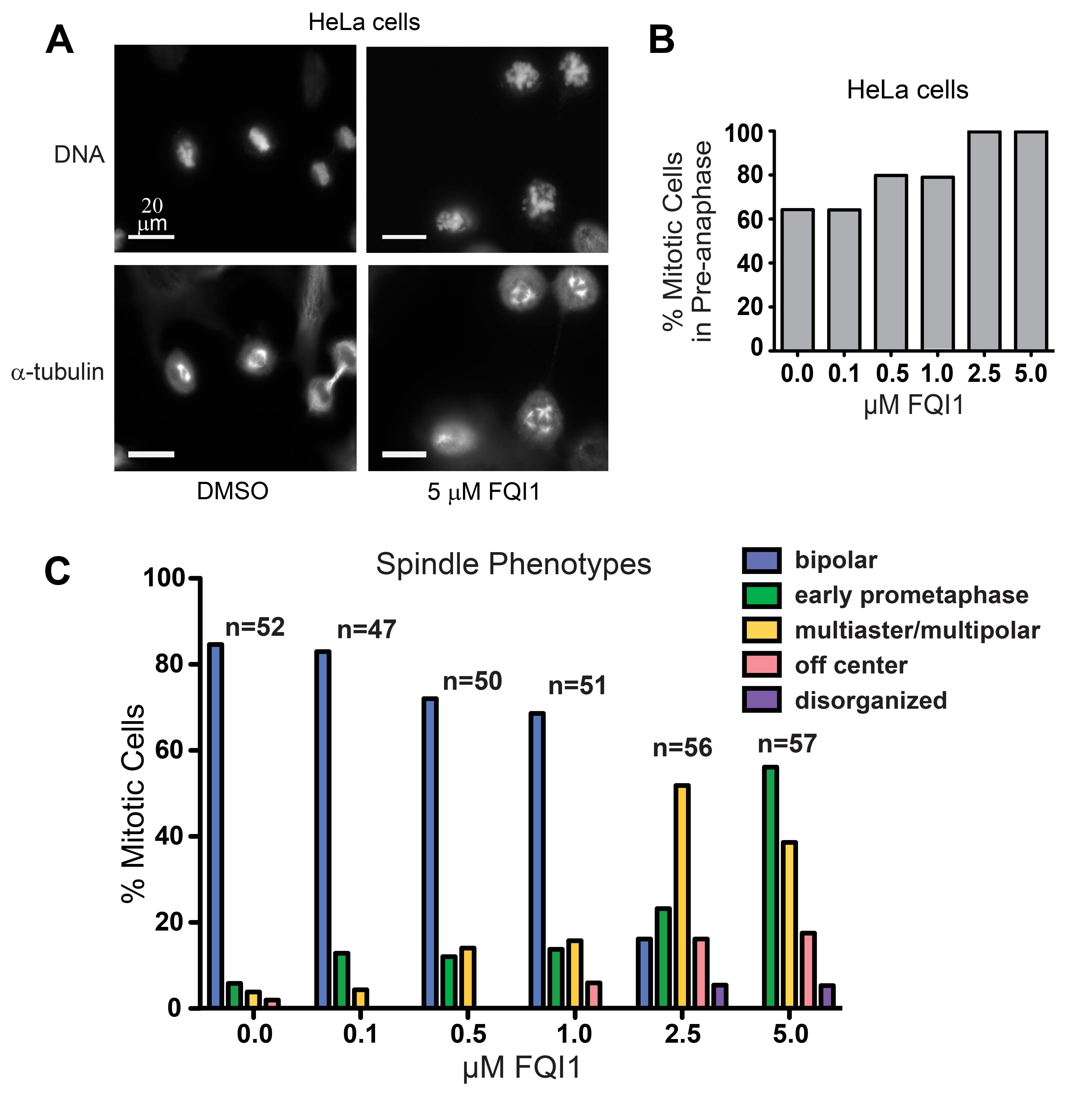

Supplement: S2 Fig — (A) Immunofluorescence of DNA and α-tubulin, as indicated, in mitotic HeLa cells. Cells were synchronized at the G1/S border by a double thymidine block and released in presence of 5 μM FQI1 or vehicle (DMSO). Fixed cells were stained with α-tubulin antibody and DAPI (DNA stain). Scale bars are 20 μm. Data are representative of 2 independent biological experiments. (B-C) An asynchronous population of HeLa cells (from the Shah laboratory) were treated with increasing doses of FQI1 for 1 h. Cells were analyzed by immunofluorescence for α-tubulin, γ-tubulin, and DNA. The mitotic index was similar amongst all the treatment groups, varying from 3–6%. (B) Each treatment group was analyzed for the percentage of mitotic cells that were pre- versus post-anaphase. (C) Mitotic spindles were classified for all mitotic cells: normal, bipolar cells, early prometaphase cells (also including early mitotic cells in which the centrosome were not significantly separated), and multipolar/multiaster cells. In the 2.5 μM FQI1 and the 5 μM FQI1 samples, there was also a general loss of α-tubulin staining. (TIF) [file pone.0268857.s002.tif]

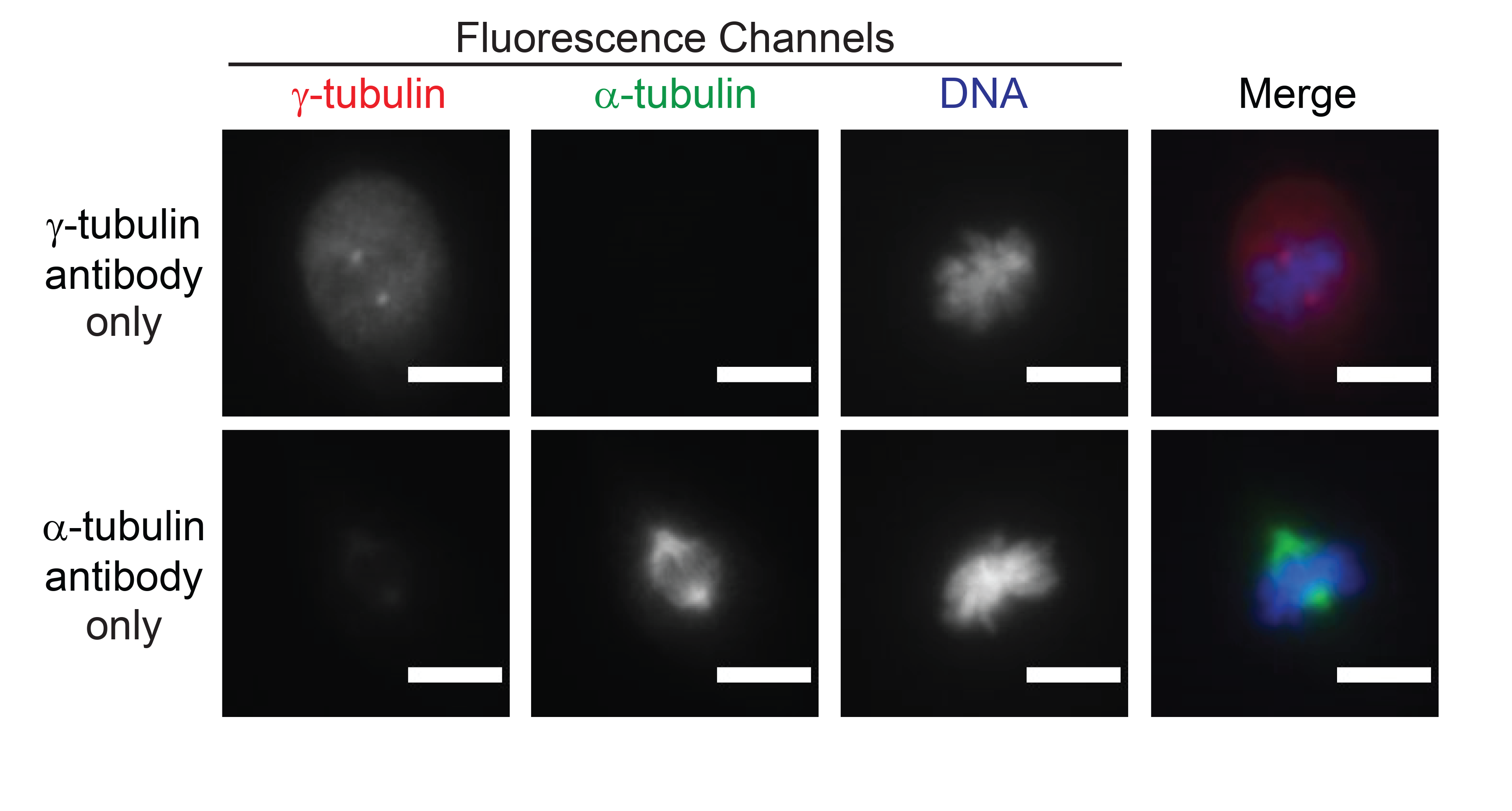

Supplement: S3 Fig — Immunofluorescence images of RPE cells treated as in Fig 4A, but stained for DNA and only one primary antibody, against either γ-tubulin (top) or α-tubulin (bottom), plus its respective secondary antibody. These images are representative from a total of 43–45 imaged cells across three independent biological experiments. Scale bars are 8 μm. These data verify the extremely minimal, if any, fluorescent emission bleed-through between the channels detecting the Alexa Fluor 546 and Cy5 fluorophores. (TIF) [file pone.0268857.s003.tif]

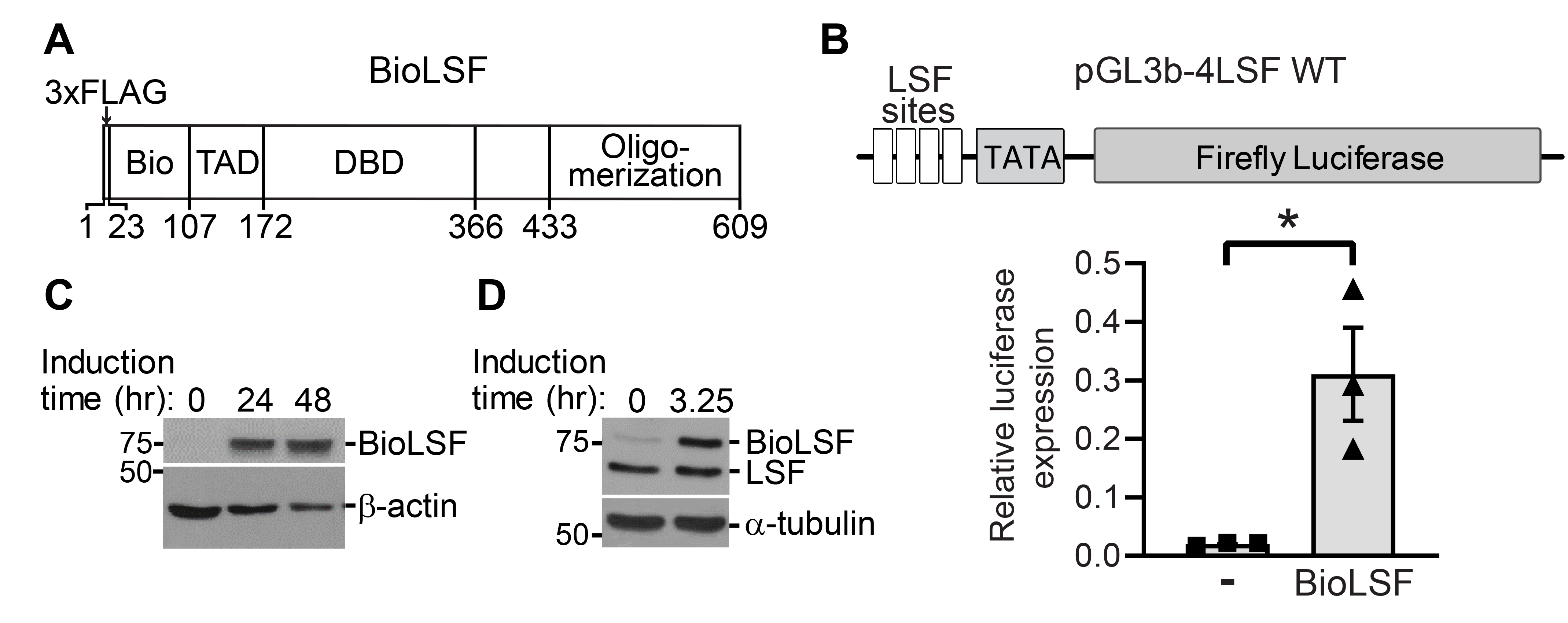

Supplement: S4 Fig — (A) Structure of the BioLSF fusion protein. Amino acid numbers are indicated. Bio: Biotinylated domain from BCCP; TAD: transcriptional activation domain of LSF; DBD: DNA binding domain of LSF; Oligomerization: a region encompassing both the LSF dimerization and tetramerization functions. (B) Dual-luciferase reporter assay to measure LSF transcriptional activity. Top: Schematic of the LSF-dependent firefly luciferase reporter construct. Bottom: Reporter activity of transfection of a BioLSF-expressing construct (BioLSF) compared to an empty vector control (-). Relative activity indicates the levels of LSF-regulated firefly luciferase activity normalized to that of the control Renilla luciferase activity. Firefly luciferase activity was increased 15-fold upon induction of BioLSF expression. Data points indicate averages of technical replicates from three independent biological experiments. Bars represent means ± SEM. Unpaired t-test, *p = 0.022. (C) Top: Streptavidin blot showing robust induction of biotinylated BioLSF upon doxycycline treatment for 24 and 48 hours of DLD-1 derived cells. Bottom: Blot for β-actin, as a loading control. Molecular weight markers are in kDa. Representative of at least three experiments. (D) Top: Blot using LSF antibody showing expression of BioLSF and LSF in DLD-1 derived lysates from uninduced and induced cells treated with doxycycline for 3.25 hours. Note the significant induction, equivalent to levels of endogenous LSF by 3.25 hours after induction. Also, note that in the tetracycline-free media, there remains some low level expression of BioLSF. Bottom: Blot for α-tubulin, as a loading control. (TIF) [file pone.0268857.s004.tif]

Figure 5C, middle panel

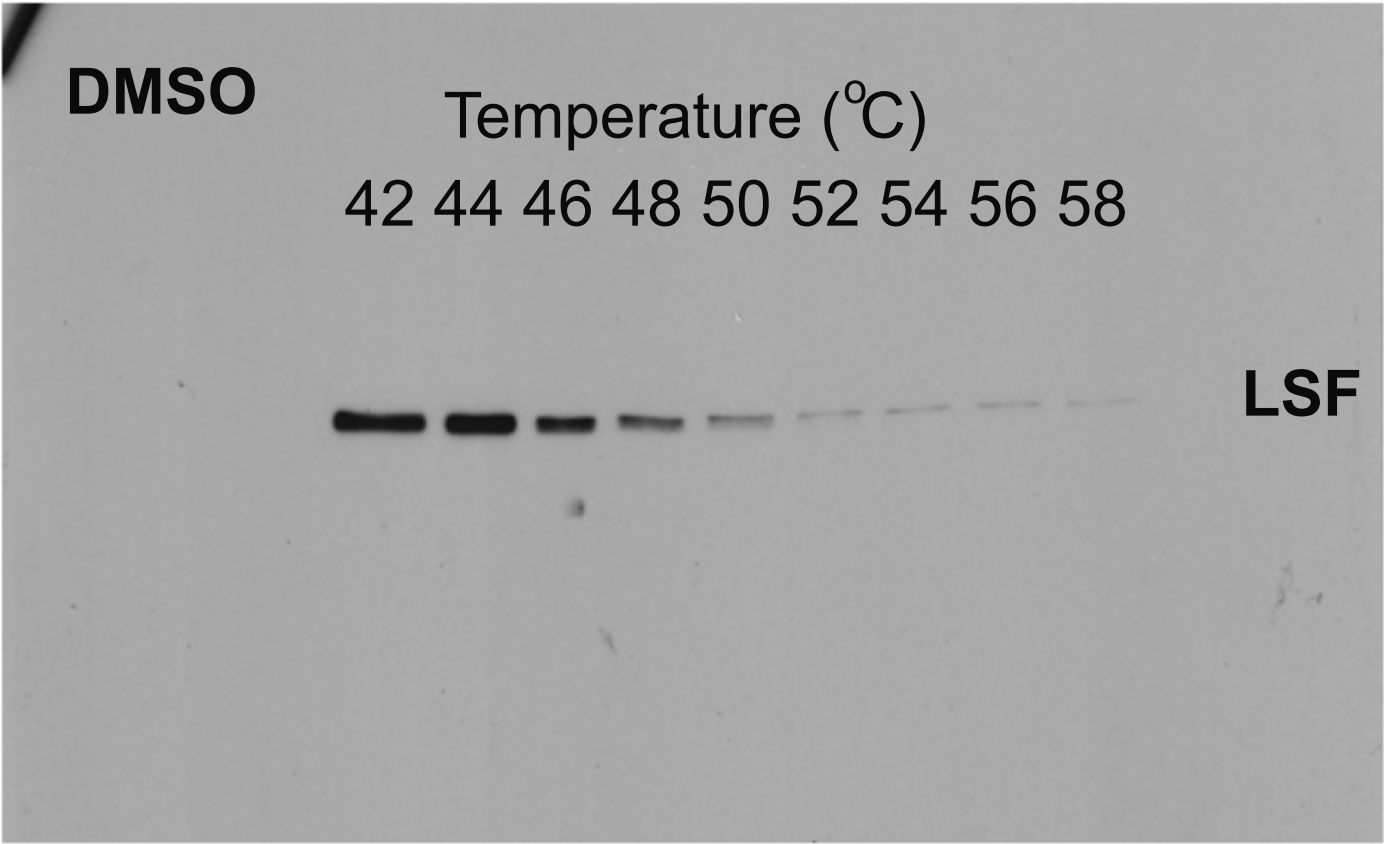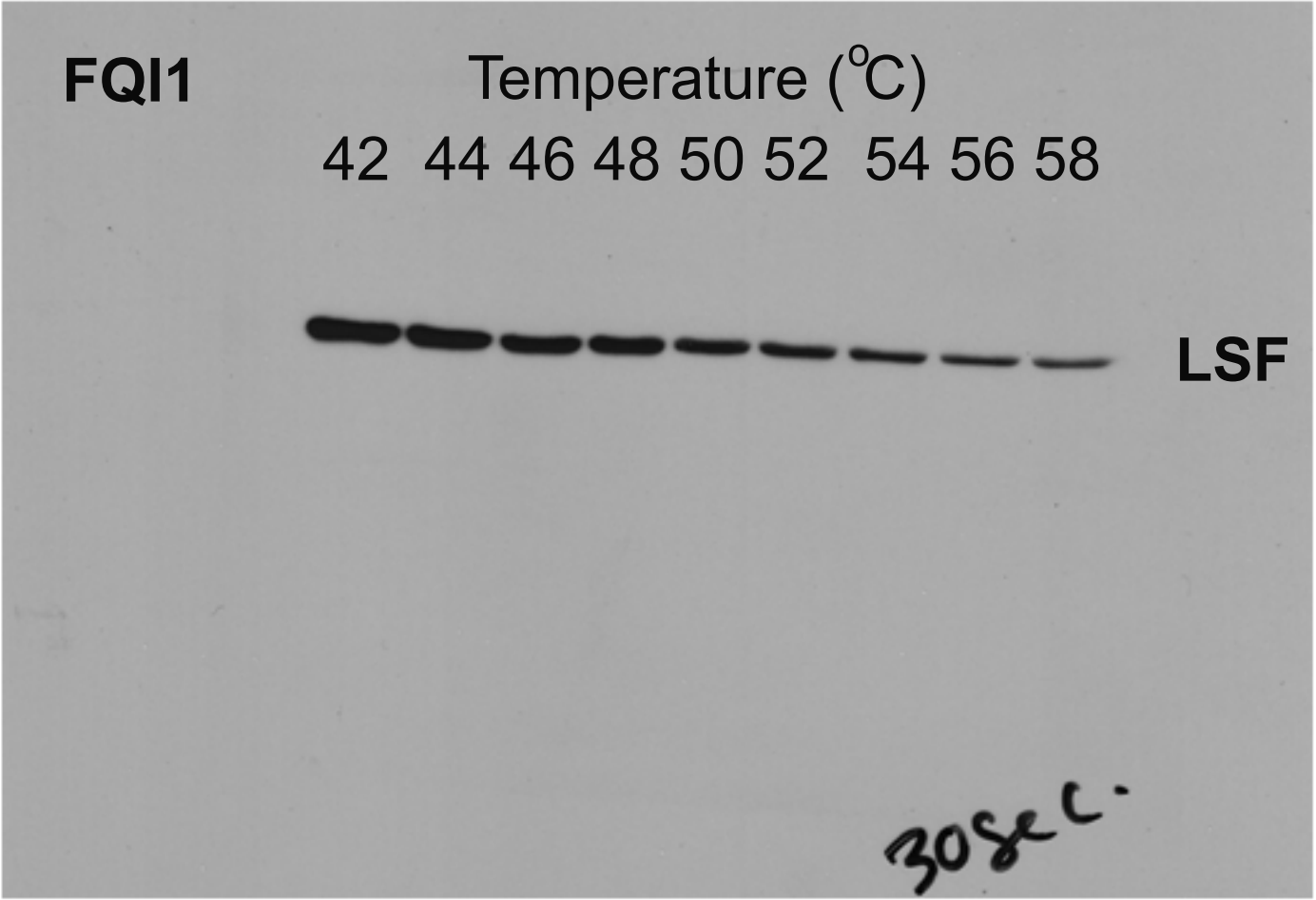

Figure 5C, right panel

DMSO

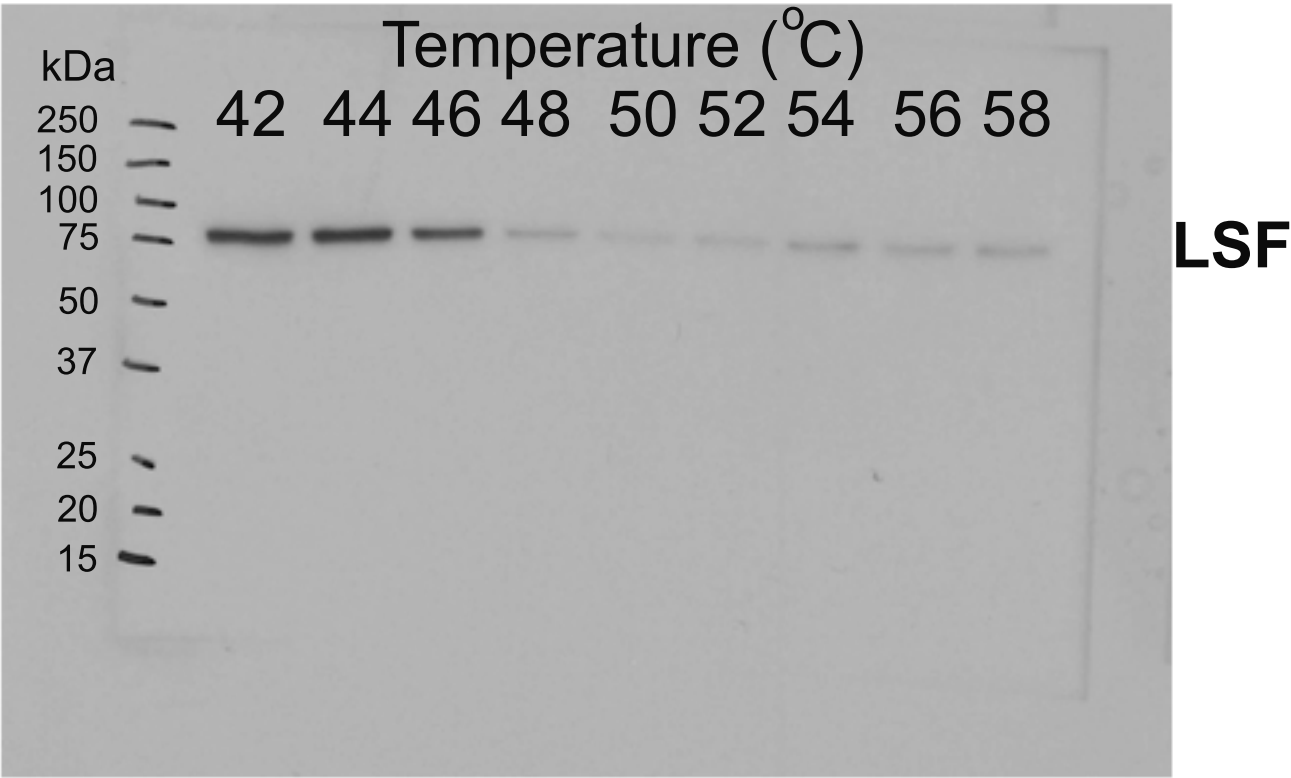

FQI34

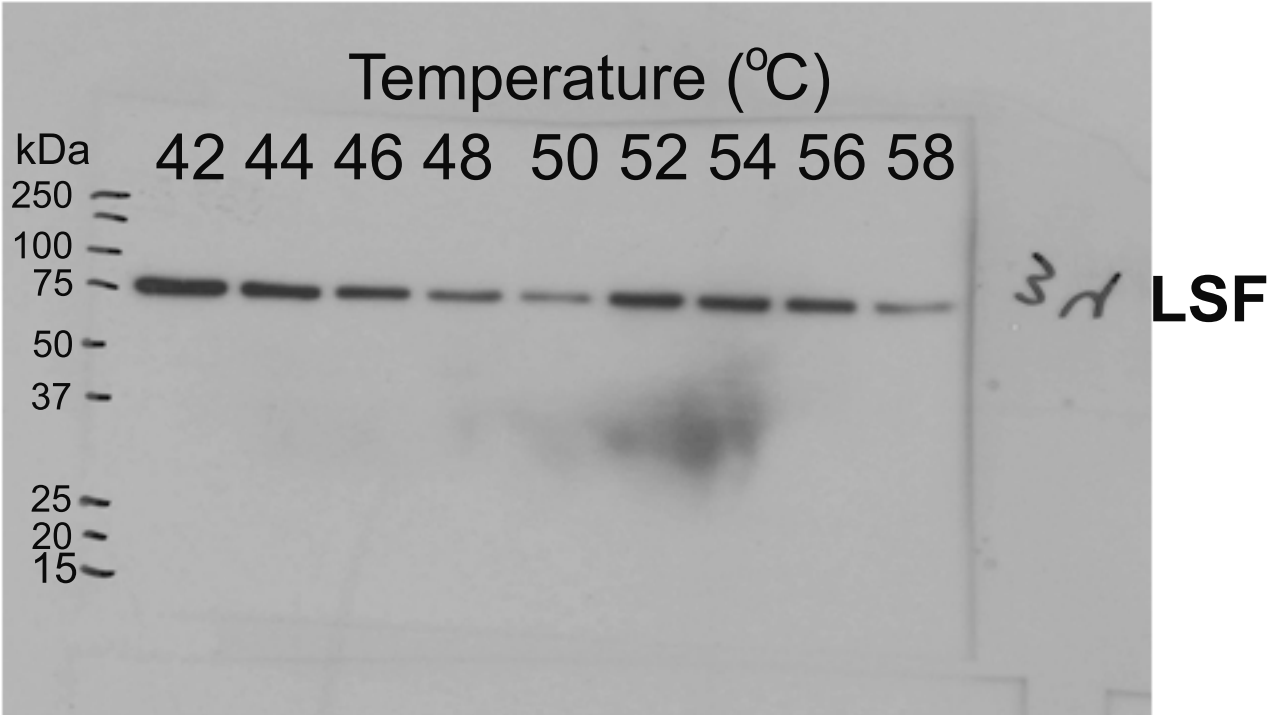

S4 FIG, panel C

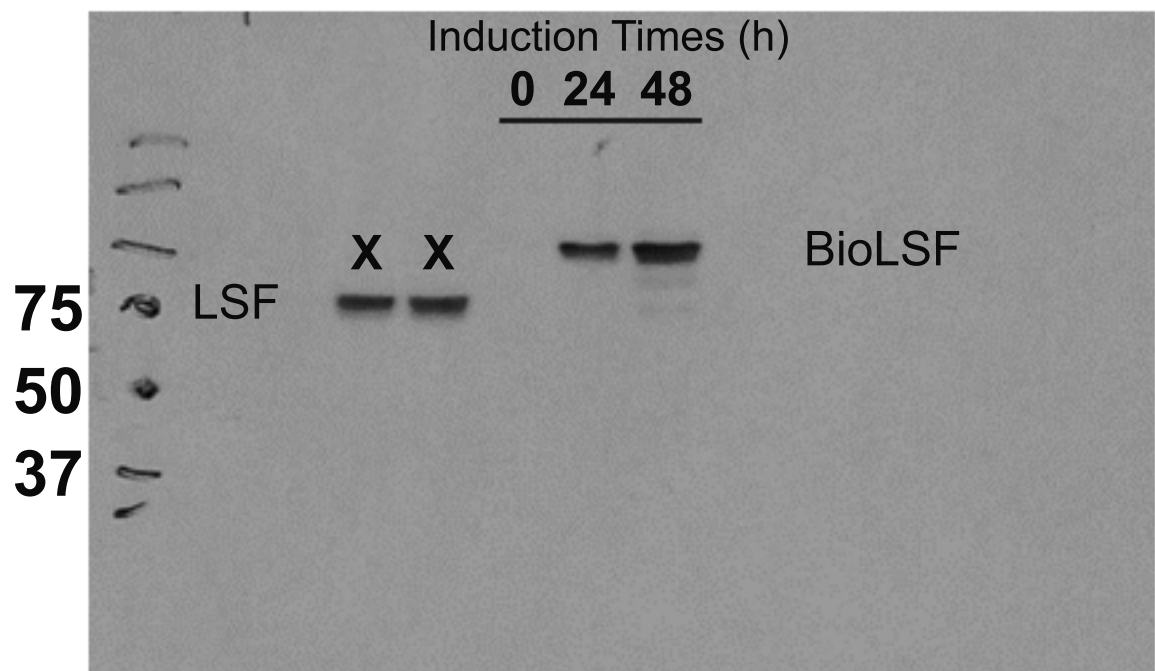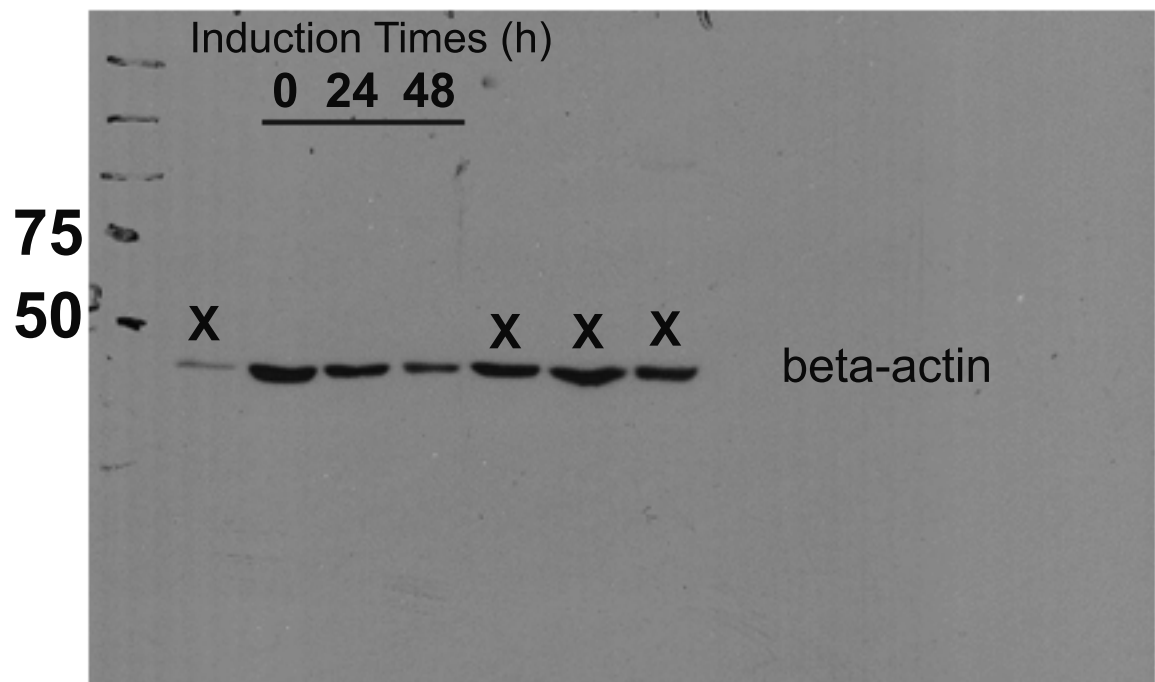

## S4 FIG, panel D

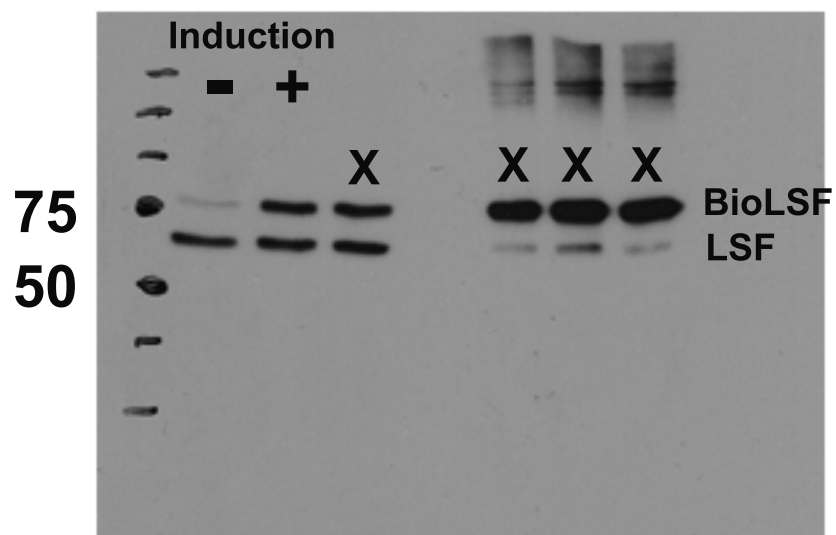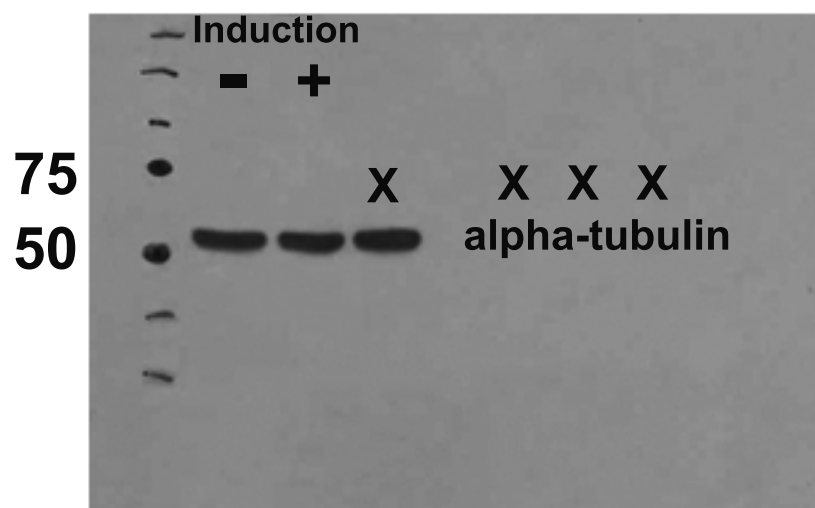

Supplement: S1 Raw images — (PDF) [file pone.0268857.s010.pdf]
