# Supplementary material for: Factor quinolinone inhibitors disrupt spindles and multiple LSF (TFCP2)-protein interactions in mitosis, including with microtubule-associated proteins
Source: PLoS One. 2022 Jun 15;17(6):e0268857. doi: 10.1371/journal.pone.0268857 (PMC9200292; doi:10.1371/journal.pone.0268857)
Supplement: S2 Table — (PDF) [file pone.0268857.s006.pdf]

Factor Quinolinone Inhibitors disrupt spindles and multiple LSF (TFCP2)-protein interactions in mitosis, including with microtubule-associated proteins

SA Yunes, JLS Willoughby, JH Kwan, JM Biagi, N Pokharel, HG Chin, EA York, K-C Su, K George, JV Shah, A Emili, SE Schaus, and U Hansen*

S2 Table. Gene Ontology analysis of complete list of mitotic BioLSF-interacting proteins.

Gene Ontology Biological Process (Direct)	%	Count	Benjamini	Gene Names
Cell-cell adhesion	14.8	62	4.2E-38	TES, DDX3X, OLA1, HDLBP, BZW1, RPL6, SEPT9, ATIC, CAPZB, KIF5B, RUVBL1, LRRFIP1, EIF2A, NUDC, ARHGEF16, RANGAP1, CKAP5, SND1, SEPT2, ATXN2L, CTTN, EEF1D, TAGLN2, MAPRE1, LRRC59, PDXDC1, USO1, IQGAP1, CNN3, PDLIM1, CNN2, DNAJB1, LIMA1, PUF60, PCBP1, MYO6, EIF4H, RPL14, PACSIN2, EPS8L2, RPL15, PDLIM5, SPTBN1, VASP, RANBP1, STAT1, IDH1, RAB11B, TJP1, EHD1, RSL1D1, MYO1B, EIF5, EHD4, FASN, CAPZA1, SERBP1, EIF3E, EIF4G2, EIF4G1, TJP2, HSPA1A
Translational initiation	6.9	29	2.7E-15	DDX3Y, RPL5, RPL6, RPL14, EIF4H, EIF3CL, RPL15, RPS11, RPS10, EIF4B, EIF2A, RPL23, RPS5, EIF2S2, EIF2S1, RPS27, EIF5, EIF3L, EIF3I, RPL37A, EIF3G, EIF3E, EIF3F, PABPC1, EIF3C, EIF3D, EIF4G2, EIF3B, EIF4G1
mRNA splicing, via spliceosome	8.1	34	3.9E-14	SF3B2, DDX23, SRSF1, HNRNPR, USP39, PRPF8, EFTUD2, SYNCRIP, SNRPD2, SNRNP70, TRA2B, PCBP1, SNRPD3, SF3B1, HNRNPA0, SF3A1, NONO, CDC5L, PRPF40A, SRRM1, PRPF4, NUDT21, PRPF6, HNRNPUL1, PSPC1, HNRNPF, HNRNPD, GEMIN5, SNRPA1, PABPC1, HNRNPH3, SRSF6, SNRNP200, SF1
tRNA aminoacylation for protein translation	4.1	17	2.1E-13	CARS, YARS, DARS, WARS, VARS, SARS, EPRS, NARS, AIMP1, QARS, KARS, MARS, GARS, IARS, HARS, FARSA, FARSB
Regulation of translational initiation	3.6	15	1.9E-11	DDX1, EIF5, EIF3L, EIF3I, EIF4H, EIF3G, EIF3CL, EIF3E, EIF3F, EIF3C, EIF3D, EIF4B, EIF4G2, EIF3B, EIF4G1
Formation of translation preinitiation complex	2.9	12	5.3E-10	EIF5, EIF3L, EIF3I, EIF4H, EIF3G, EIF3CL, EIF3E, EIF3F, EIF3C, EIF3D, EIF4B, EIF3B
RNA splicing	5.5	23	3.1E-08	RBM39, SF3B2, NONO, DDX23, USP39, PRPF8, SUPT6H, CCAR2, SRRM1, EFTUD2, PRPF4, SYNCRIP, PRPF6, SNRPD2, PUF60, SNRNP70, PPP2R1A, THRAP3, SNRPA1, SNRPD3, HNRNPH3, TARDBP, ZPR1

mRNA processing	5.5	23	1.2E-07	KHDRBS1, RBM39, SF3B2, SF3A1, NONO, SRSF1, HNRNPR, HNRNPLL, USP39, PRPF8, SUPT6H, CCAR2, SRRM1, EFTUD2, NUDT21, PUF60, SNRNP70, THRAP3, PDE12, XRN2, TARDBP, HNRNPA0, ZPR1
Cell division	7.4	31	5.8E-07	NUMA1, VPS4A, CLTC, KIF11, USP39, SMC3, NCAPH, AURKA, PPP1CB, SEPT9, TPR, RUVBL1, BUB3, MAP4, MISP, PELO, SEPT11, NUDC, ANXA11, PRPF40A, ATAD3B, SMC1A, CKAP5, SEPT2, CD2AP, PPP1CA, KIFC1, CDC37, CDK1, KIF2C, MAPRE1
Regulation of mRNA stability	3.8	16	5.6E-06	PSMD11, PSMC5, XPO1, PSMA1, PSMD5, PSME3, PSMC2, PSMD3, SERBP1, HNRNPD, PSME1, PABPC1, TNPO1, HSPA1B, HSPA1A, EIF4G1
Nuclear-transcribed mRNA catabolic process, nonsense-mediated decay	4.1	17	5.6E-06	RPL5, UPF1, RPL23, RPS5, PPP2R2A, RPL6, RPS27, PPP2R1A, RPL37A, RPL14, ETF1, EIF3E, PABPC1, RPL15, RPS11, RPS10, EIF4G1
Regulation of cellular response to heat	3.1	13	3.9E-05	CAMK2B, CAMK2D, CAMK2A, CCAR2, NUP93, DNAJB1, HSPH1, DNAJC7, TPR, FKBP4, CAMK2G, HSPA1B, HSPA1A
Translation	4.8	20	2.3E-03	RPL5, DARS, WARS, RPL23, RPS5, SARS, RRBP1, RPL6, EFTUD2, RSL1D1, RPS27, RPL37A, RPL14, RPL15, HARS, RPS11, RPS10, PELO, FARSB, EIF4G1
Anaphase-promoting complex-dependent catabolic process	2.6	11	2.7E-03	PSMC5, PSMD11, PSMA1, PSMD5, PSME3, PSMC2, PSMD3, CDK1, PSME1, BUB3, AURKA
RNA splicing, via transesterification reactions	1.7	7	2.7E-03	PRPF4, PRPF6, TRA2B, DDX23, PRPF8, SF3B1, SRRM1
Nucleobase-containing small molecule interconversion	1.7	7	2.7E-03	NME1-NME2, RRM1, TXNRD1, GSR, CMPK1, NME2, NME1
RNA processing	2.9	12	2.7E-03	RBM39, PRPF4, SYNCRIP, SF3A1, HNRNPUL1, HNRNPF, XRN2, HNRNPD, HNRNPH3, SNRPD3, U2SURP, CHERP
Spliceosomal complex assembly	1.7	7	3.2E-03	PRPF6, SNRPD2, DDX1, SNRPD3, USP39, SF3B1, SF1
Viral process	5.0	21	4.9E-03	RANBP1, FDPS, USP7, SF3B2, HDAC1, COPB1, SUPT6H, SND1, IPO5, NUP93, SYNCRIP, RAD50, XPO1, KARS, PSMC2, TPR, EIF4H, ZYX, TNPO1, TLN1, EIF4G1
Negative regulation of ubiquitin-protein ligase activity involved in mitotic cell cycle	2.4	10	5.1E-03	PSMC5, PSMD11, PSMA1, PSMD5, PSME3, PSMC2, PSMD3, CDK1, PSME1, BUB3

Intracellular protein transport	4.3	18	6.5E-03	VPS29, COPA, COPB1, USO1, CLTC, AP3B1, AP2B1, IPO4, IPO5, ARCN1, EHD1, XPO1, CTTN, MYO6, VPS35, COPG1, TNPO1, YWHAH
Viral transcription	2.9	12	7.7E-03	NUP93, RPL5, RPS27, RPS5, RPL23, TPR, RPL37A, RPL14, RPL15, RPS11, RPS10, RPL6
Positive regulation of ubiquitin-protein ligase activity involved in regulation of mitotic cell cycle transition	2.4	10	7.7E-03	PSMC5, PSMD11, PSMA1, PSMD5, PSME3, PSMC2, PSMD3, CDK1, PSME1, BUB3
Mitotic nuclear division	4.3	18	1.0E-02	NUDC, PPP1R12A, NUMA1, CLTC, KIF11, SMC3, CKAP5, SEPT2, AURKA, CD2AP, TUBB3, TPR, RUVBL1, CDK1, KIF2C, SUGT1, MAPRE1, MISP

[Total: 388 protein groups including 418 proteins; Benjamini-Hochberg adjusted p-value ≤ 0.01]
