# Supplementary material for: Factor quinolinone inhibitors disrupt spindles and multiple LSF (TFCP2)-protein interactions in mitosis, including with microtubule-associated proteins
Source: PLoS One. 2022 Jun 15;17(6):e0268857. doi: 10.1371/journal.pone.0268857 (PMC9200292; doi:10.1371/journal.pone.0268857)
Supplement: S3 Table — (PDF) [file pone.0268857.s007.pdf]

Factor Quinolinone Inhibitors disrupt spindles and multiple LSF (TFCP2)-protein interactions in mitosis, including with microtubule-associated proteins

SA Yunes, JLS Willoughby, JH Kwan, JM Biagi, N Pokharel, HG Chin, EA York, K-C Su, K George, JV Shah, A Emili, SE Schaus, and U Hansen*

S3 Table. Gene Ontology analysis of mitotic BioLSF-interacting proteins reduced by FQI1.

Gene Ontology Biological Process (Direct)	%	Count	Benjamini	Gene Names
Cell-cell adhesion	13.3	17	1.5E-07	VASP, RANBP1, PDXDC1, ARHGEF16, HDLBP, RANGAP1, CKAP5, RAB11B, ATXN2L, RSL1D1, LIMA1, MYO1B, PUF60, MYO6, RUVBL1, RPL14, EIF2A
mRNA splicing, via spliceosome	10.2	13	4.3E-05	SF3B2, DDX23, SRSF1, HNRNPR, USP39, SRRM1, EFTUD2, PRPF4, PRPF6, SNRPD2, GEMIN5, SNRPA1, SF1
RNA splicing	8.6	11	1.3E-04	EFTUD2, PRPF4, SF3B2, PRPF6, SNRPD2, PUF60, DDX23, SNRPA1, USP39, SUPT6H, SRRM1
mRNA processing	7.8	10	1.6E-03	EFTUD2, SF3B2, PUF60, PDE12, SRSF1, HNRNPR, USP39, HNRNPLL, SUPT6H, SRRM1
Cell division	10.2	13	2.0E-03	SEPT11, SMC1A, ATAD3B, USP39, CKAP5, NCAPH, AURKA, PPP1CA, TPR, RUVBL1, CDK1, KIF2C, PELO
Spliceosomal complex assembly	3.9	5	5.5E-03	PRPF6, SNRPD2, DDX1, USP39, SF1

[Total 125 protein groups including 128 proteins; Benjamini-Hochberg adjusted p-value ≤ 0.01]
