# Supplementary material for: Factor quinolinone inhibitors disrupt spindles and multiple LSF (TFCP2)-protein interactions in mitosis, including with microtubule-associated proteins
Source: PLoS One. 2022 Jun 15;17(6):e0268857. doi: 10.1371/journal.pone.0268857 (PMC9200292; doi:10.1371/journal.pone.0268857)
Supplement: S4 Table — (PDF) [file pone.0268857.s008.pdf]

Factor Quinolinone Inhibitors disrupt spindles and multiple LSF (TFCP2)-protein interactions in mitosis, including with microtubule-associated proteins

SA Yunes, JLS Willoughby, JH Kwan, JM Biagi, N Pokharel, HG Chin, EA York, K-C Su, K George, JV Shah, A Emili, SE Schaus, and U Hansen*

Table S4.
Gene Ontology analysis of mitotic BioLSF-interacting proteins enhanced by FQI1.

Gene Ontology Biological Process (Direct)	%	Count	Benjamini	Gene Names
Arp2/3 complex-mediated actin nucleation	8	4	0.013	ACTR2, ARPC2, ARPC4-TTLL3, ARPC4

[Total 48 protein groups including 50 proteins; Benjamini-Hochberg adjusted p-value ≤ 0.05]
