# Supplementary material for: Factor quinolinone inhibitors disrupt spindles and multiple LSF (TFCP2)-protein interactions in mitosis, including with microtubule-associated proteins
Source: PLoS One. 2022 Jun 15;17(6):e0268857. doi: 10.1371/journal.pone.0268857 (PMC9200292; doi:10.1371/journal.pone.0268857)
Supplement: S1 File — (PDF) [file pone.0268857.s009.pdf]

Factor Quinolinone Inhibitors disrupt spindles and multiple LSF (TFCP2)-protein interactions in mitosis, including with microtubule-associated proteins

Sarah A. Yunes, Jennifer L.S. Willoughby, Julian H. Kwan, Jessica M. Biagi, Niranjana Pokharel, Hang Gyeong Chin, Emily A. York, Kuan-Chung Su, Kelly George, Jagesh V. Shah, Andrew Emili, Scott E. Schaus, and Ulla Hansen*

Preparation and characterization of reagents:
FQIs, DLD-1 derived cell line, and BioLSF

Table of Contents

Synthesis and characterization of FQI34 2
Cell proliferation/viability assays: FQI1 and FQI34 6
Cell thermal stability assays (CETSAs): FQI1 and FQI34 7
Dual luciferase reporter assays: FQI1 and FQI34 7
Generation of the DLD-1 Flp-In™ T-REx™ TIR1 BioLSF (DLD-1 derived) cell line 8
Characterization of BioLSF activity: Luciferase reporter assay 8
Streptavidin blotting and Immunoblotting 9

SUPPLEMENTAL METHODS AND RESULTS

Synthesis and characterization of FQI34

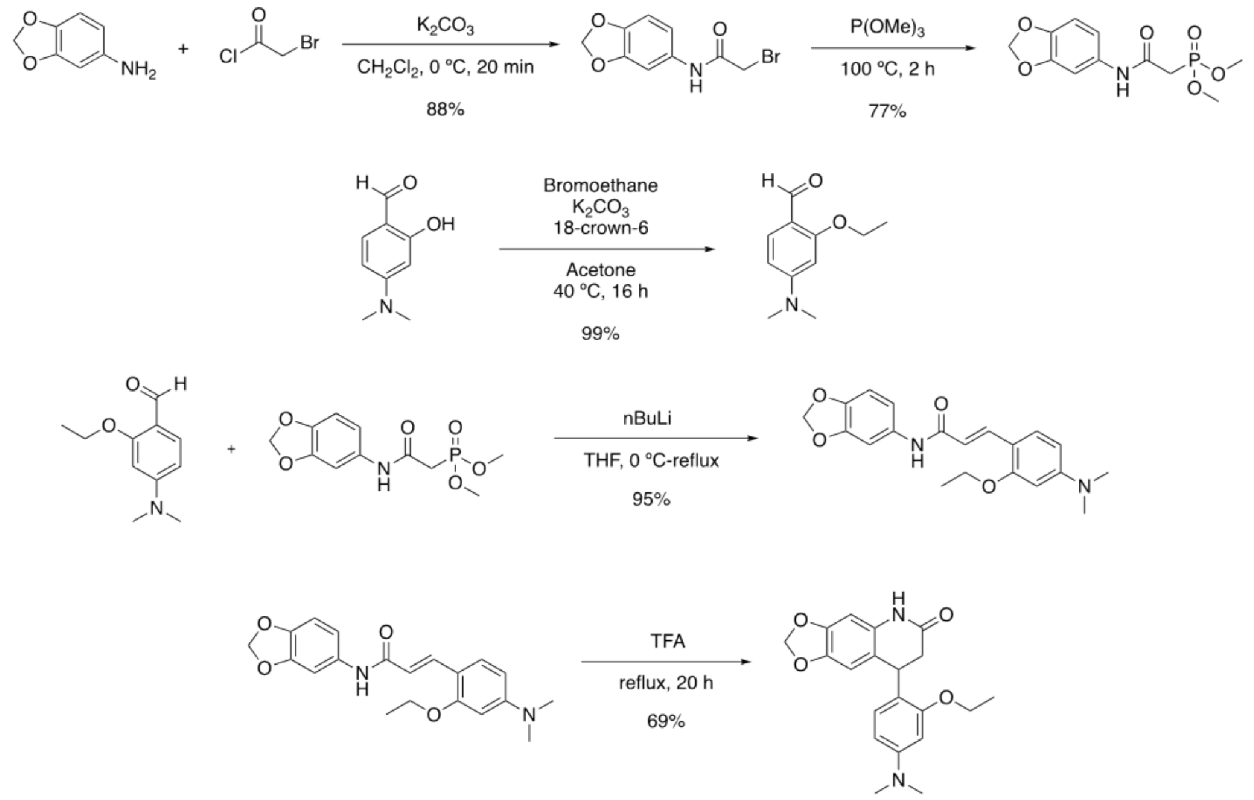

General Information. All ^{1}H NMR spectra were obtained at 400 MHz and referenced to the $CHCl_3$ singlet at 7.26 ppm, or the DMSO singlet at 2.50 ppm. ^{13}C NMR spectra were obtained at 100 MHz, and referenced to the center peak of the $CDCl_3$ triplet at 77.16 ppm, or the center of the DMSO-d_6 septet at 39.51 ppm. Chemical shifts are reported in parts per million as follows: chemical shift, multiplicity (s = singlet, d = doublet, t = triplet, q = quartet, m = multiplet), coupling constant, and integration. High resolution mass spectrometry data were obtained on a Waters Qtof (hybrid quadrupolar/time-of-flight) API US system by electrospray (ESI) in the positive mode. Mass correction was done by an external reference using a Waters Lockspray accessory. Mobile phases were water and acetonitrile with 0.1% formic acid. The MS settings were: capillary voltage = 3kV, cone voltage = 35, source temperature = 120 °C and dissolvation temperature = 350 °C. Also, we are grateful to the National Science Foundation for the purchase of the Waters high resolution mass spectrometer (CHE 0443618) used in this work, and the Boston University Chemistry Department Chemical Instrumentation Center for acquiring the HRMS data. Flash column chromatography was performed on Sorbent Technologies 60 Å silica gel. FQI-34 was

prepared according to the patented procedures.[1-2]

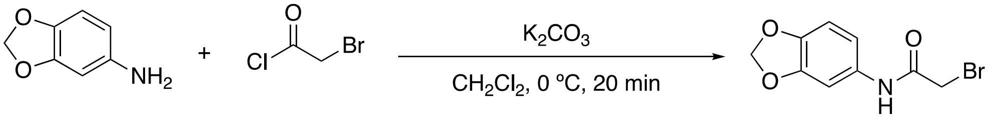

N-(benzo[d][1,3]dioxol-5-yl)-2-bromoacetamide. A flame-dried 500-mL round bottomed flask equipped with a Teflon-coated magnetic stirbar under an argon atmosphere was charged with 3,4-(methylenedioxy)aniline (5.88 g, 42.9 mmol) and dry dichloromethane (100 mL, 0.43 M). (Note: 3,4-(methylenedioxy)aniline was recrystallized from hexanes prior to use). Vacuum oven-dried potassium carbonate (8.30 g, 60 mmol, 1.40 equiv) was added, and the reaction was cooled to 0 ºC. Bromoacetyl chloride (4.64 mL, 55.7 mmol, 1.30 equiv) was added *via* syringe and the reaction was stirred at 0 ºC for 20 min and then allowed to warm to room temperature. Saturated aqueous sodium bicarbonate was added (100 mL) and the mixture was extracted with dichloromethane (3 x 150 mL). The combined organic layers were washed with saturated aqueous sodium chloride (100 mL), and dried over anhydrous sodium sulfate (Na_2SO_4). The filtrate was concentrated *via* rotary evaporation to afford N-(benzo[d][1,3]dioxol-5-yl)-2-bromoacetamide as a tan solid (9.73 g, 37.7 mmol, 88% yield, >97% purity), that was used without purification. ^{1}H NMR (DMSO-d_6, 400 MHz) δ 10.30 (s, 1H), 7.27 (d, J = 2.0 Hz, 1H), 6.96 (dd, J = 8.4, 2.0 Hz, 1H), 6.87 (d, J = 8.4 Hz, 1H), 5.98 (s, 2H), 4.00 (s, 2H). ^{13}C NMR (DMSO-d_6, 100 MHz) δ 164.4, 147.1, 142.9, 133.0, 112.2, 108.1, 101.3, 101.1, 30.4. HRMS *m/z* 257.9771 [(M + H^+) calculated for $C_9H_9BrNO_3^+$: 257.9766].

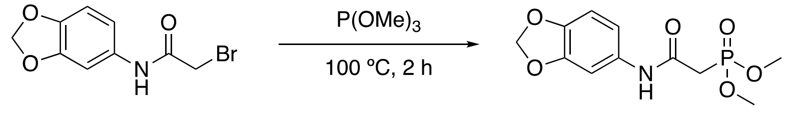

Dimethyl (2-(benzo[d][1,3]dioxol-5-ylamino)-2-oxoethyl)phosphonate. A flame dried 50-mL round-bottomed flask equipped with a Teflon-coated magnetic stirbar under an argon atmosphere was charged with N-(benzo[d][1,3]dioxol-5-yl)-2-bromoacetamide (4.00 g, 15.5 mmol) and

[1] Hansen, U.; Schaus, S.; Grant, T.; Bishop, J.; Kavouris, J.; Christadore, L. M.; Inhibitors of Late SV40 Factor (LSF) as Cancer Chemotherapeutics. U.S. Patent 9,815,845 B2, August 27, 2019.

[2] Schaus, S. E.; Hansen, U.; York, E.A.; Pokharel, N.; Quinolin-2(1h)-one Inhibitors Of Late SV40 Factor. U.S. Provisional Application No. 63/073,240, filed September 1, 2020.

trimethyl phosphite (9.62 g, 9.16 mL, 77.5 mmol). The flask was fitted with a reflux condenser, rubber septum, and argon balloon. The reaction was heated to 100 °C for 2 hours, at which time the reaction was poured into a 500-mL separatory funnel, diluted with dichloromethane (250 mL), and washed with water (3 x 200 mL). The organic layer was rinsed with saturated aqueous sodium chloride (50 mL), and dried over anhydrous sodium sulfate (Na_2SO_4). The filtrate was concentrated in vacuo to afford a viscous oil that was left under high vacuum (0.4 mmHg) overnight. Then, the product was dissolved in dry toluene (50 mL) and concentrated *via* rotary evaporation to azeotrope any remaining trimethyl phosphite. The product was further dried under high vacuum (0.4 mmHg) to afford dimethyl (2-(benzo[d][1,3]dioxol-5-ylamino)-2-oxoethyl)phosphonate as a pale pink/purple solid (3.43 g, 77% yield, >98% purity) that was used without further purification. ^{1}H NMR ($CDCl_3$, 400 MHz) δ 8.89 (s, 1H), 7.18 (d, J = 2.1 Hz, 1H), 6.79 (dd, J = 8.4, 2.1 Hz, 1H), 6.65 (d, J = 8.4 Hz, 1H), 5.91 (s, 2H), 3.82 (d = 11.2 Hz, 6H), 3.01 (d, J = 21.0 Hz, 2H). ^{13}C NMR ($CDCl_3$, 100 MHz) δ 161.7, 147.5, 144.0, 132.3, 112.8, 107.8, 102.4, 53.4, 35.9, 34.6. HRMS *m/z* 288.0630 [(M + H$^+$) calculated for $C_{11}H_{15}BrNO_6P^+$: 288.0637].

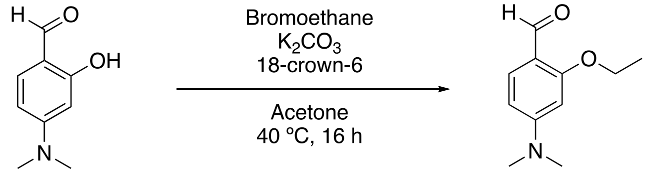

4-(dimethylamino)-2-ethoxybenzaldehyde. A flame-dried 250-mL round-bottomed flask equipped with a Teflon-coated magnetic stirbar under an argon atmosphere was charged with 4-(dimethylamino)-2-hydroxy-benzaldehye (4.13 g, 25 mmol) in acetone (83 mL, 0.30 M). Anhydrous potassium carbonate (5.18 g, 37.5 mmol), 18-crown-6 (330 mg, 1.25 mmol) and bromoethane (13.6 g, 125 mmol, 9.27 mL) were added to the reaction. The reaction mixture was fitted with a reflux glycol condenser and heated at 40 ºC for 16 hours, then cooled to room temperature. The reaction was filtered and the filtered solid was washed with acetone. The filtrate was evaporated *via* rotary evaporation to yield 4-(dimethylamino)-2-ethoxy-benzaldehyde as a brown solid (3.87g, >99% yield, 90% pure) that was used without further purification. ^{1}H NMR ($CDCl_3$, 400 MHz) δ 10.20 (s, 1H), 7.72 (d, J = 8.8 Hz, 1H), 6.29 (dd, J = 8.8, 2.3 Hz, 1H), 6.02 (d, J = 2.3 Hz, 1H), 4.11 (q, J = 7.0 Hz, 2H), 3.06 (s, 6H), 1.46 (t, J = 7.0 Hz, 3H). ^{13}C NMR ($CDCl_3$, 100 MHz) 187.6, 163.4, 155.9, 129.8, 114.6, 104.50, 93.7, 70.0, 63.7, 40.2, 14.7. HRMS *m/z* 194.1176 [(M + H$^+$) calculated for $C_{11}H_{16}NO_2^+$: 194.1181].

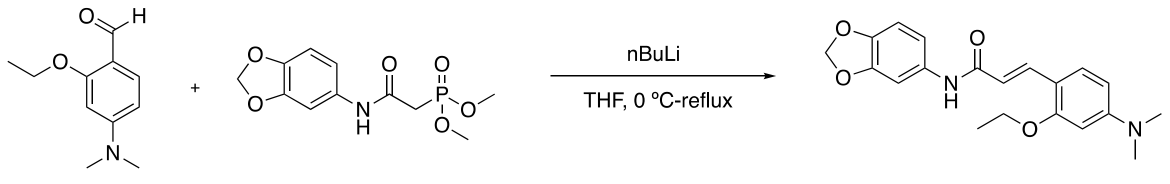

(E)-N-(benzo[d][1,3]dioxol-5-yl)-3-(4-(dimethylamino)-2-ethoxyphenyl)acrylamide. A flame dried 25-mL round-bottomed flask equipped with a Teflon-coated magnetic stirbar was charged with dimethyl (2-(benzo[d][1,3]dioxol-5-ylamino)-2-oxoethyl)phosphonate (718 mg, 2.50 mmol) and THF (8.3 mL, 0.30 M). The flask was flushed with argon and fitted with a rubber septum and argon balloon, then cooled to 0 °C in an ice-water bath. n-Butyllithium (1.6 M in hexanes, 1.35 equiv) was added dropwise, and the mixture was allowed to warm to RT and stir for 30 min, at which time 4-(dimethylamino)-2-ethoxybenzaldehyde (387 mg, 2.0 mmol) was added as a single portion. The flask was fitted with a reflux condenser and argon balloon, and the mixture was heated to reflux for 22 h. The mixture was cooled to RT and quenched with saturated ammonium chloride (5 mL). The mixture was transferred to a 500-mL separatory funnel, and diluted with dichloromethane (150 mL) and water (50 mL). The dichloromethane layer was removed and washed with water (3 x 50 mL), then dried over anhydrous sodium sulfate (Na_2SO_4), filtered, and concentrated by rotary evaporation. The product was isolated as a yellow brown solid (671 mg, 95% yield, 5:1 E:Z) and used without further purification. ^{1}H NMR ($CDCl_3$, 400 MHz) δ 7.90 (d, J = 15.3 Hz, 1H), 7.07 (s, 1H), 7.35 (d, J = 8.2 Hz, 1H), 6.84 (d, J = 8.2 Hz, 1H), 6.73 (d, J = 8.5 Hz, 1H), 6.44 (d, J = 15.3 Hz, 1H), 6.27 (dd, J = 8.5, 2.4 Hz, 1H), 6.14 (d, J = 2.4 Hz, 1H), 5.93 (s, 2H), 4.09 (q, J = 6.9 Hz, 2H), 3.00 (s, 6H), 1.47 (t, J = 6.9 Hz, 3H). ^{13}C NMR ($CDCl_3$, 100 MHz) 165.9, 159.7, 152.7, 147.6, 138.0, 135.2, 133.2, 130.3, 115.8, 112.8, 112.2, 108.0, 104.6, 102.7, 101.1, 97.2, 95.5, 63.7, 40.3, 14.9. HRMS *m/z* 355.1650 [(M + H$^+$) calculated for $C_{20}H_{23}N_2O_4^+$: 355.1650].

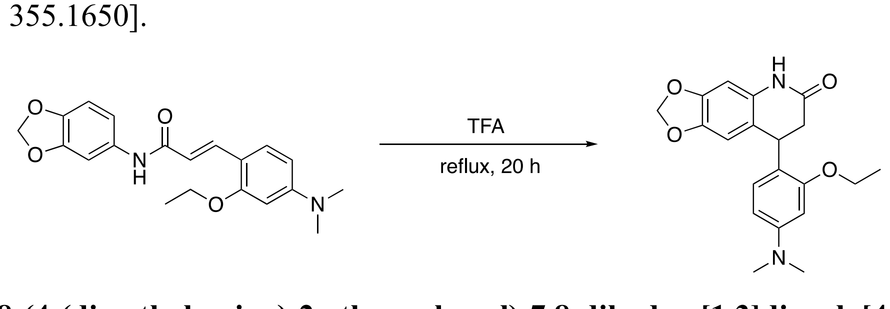

FQI-34 (8-(4-(dimethylamino)-2-ethoxyphenyl)-7,8-dihydro-[1,3]dioxolo[4,5-g]quinolin-6(5H)-one). A flame-dried 25-mL round-bottomed flask equipped with a Teflon-coated magnetic stirbar was charged with (E)-N-(benzo[d][1,3]dioxol-5-yl)-3-(4-(dimethylamino)-2-

ethoxyphenyl)acrylamide (580 mg, 1.64 mmol) and trifluoroacetic acid (11 mL, 0.15 M). The flask was flushed with argon, and fitted with a reflux condenser, a rubber septum, and an argon balloon and refluxed for 20 h. The resulting mixture was cooled to room temperature, and transferred to a 500-mL Erlenmeyer flask. The mixture was diluted with dichloromethane (150 mL) and cooled to 0 ºC in an ice bath. The reaction mixture was quenched with saturated aqueous sodium bicarbonate (~100 mL) and then transferred to a 500-mL separatory funnel. The organic layer was removed, and the aqueous layer was extracted with dichloromethane (2 x 50 mL). The combined organic layers were washed with saturated aqueous sodium chloride (50 mL), dried over anhydrous sodium sulfate (Na_2SO_4), filtered, and concentrated *via* rotary evaporation. The crude product was purified *via* column chromatography (gradient from hexanes to 1:1 hexanes:ethyl acetate) to afford the desired product as a pale orange-yellow solid (400 mg, 69% yield, >99% pure). ^{1}H NMR ($CDCl_3$, 400 MHz) δ 7.79 (s, 1H), 6.73 (d, J = 8.4 Hz, 1H), 6.47 (s, 1H), 6.35 (s, 1H), 6.27 (d, J = 2.4 Hz, 1H), 6.22 (J = 8.4, 2.4 Hz, 1H), 5.88 (s, 2H), 4.49 (dd, J = 7.0, 7.0 Hz, 1H), 4.05 (m, 2H), 2.94-2.87 (overlap, 7H), 2.76 (dd, J = 16.2, 6.4 Hz, 1H), 1.38 (t, 6.9 Hz, 3H). ^{13}C NMR ($CDCl_3$, 100 MHz) δ 171.8, 157.1, 151.0, 147.2, 143.9, 131.5, 128.6, 119.8, 117.8, 108.4, 104.7, 108.4, 104.7, 101.1, 97.6, 97.0, 63.4, 40.8, 37.0, 35.1, 14.9. HRMS *m/z* 355.1648 [(M + H^+) calculated for $C_{20}H_{23}N_2O_4^+$: 355.1658].

Cell proliferation/viability assays: FQI1 and FQI34

Three thousand RPE-hTERT-Flp-In (RPE) cells or DLD-1 Flp-In™ T-REx™ TIR1 cells were seeded per well in a 96-well plate and incubated at 37 °C for 20 hours. Compound dilutions were prepared in anhydrous DMSO and added to the cell culture media to a final DMSO concentration of 1% for a 72 hour treatment. Cell growth inhibition was assessed by measuring absorbance at 490 nm after an approximately 1-hour incubation using reagent from the Promega CellTiter 96® AQueous One Solution Cell Proliferation Assay (Promega, G358C), as directed. Percent growth inhibition was determined by the ratio of the absorbance of treatment wells to the absorbance of control wells. A non-linear regression plot of the percentage of growth inhibition versus compound concentration was prepared using GraphPad Prism software, using settings for the curves of variable slope and four parameters. The relative GI50 value was determined from the curve as the concentration of drug that provokes a response halfway between the top and bottom plateaus of the curve.

Cell thermal stability assays (CETSAs): FQI1 and FQI34

Huh7 cells were obtained from JCRB (Japanese Cancer Resources Bank), and cultured in DMEM medium (Dulbecco's modification of Eagle's Medium, Corning) supplemented with 10% fetal bovine serum (Gibco). The cells were cultured at 37 °C in 5% CO_2. Approximately $9x10^5$ Huh7 cells were plated per 10 cm plate. After 20 hours, cells were treated for three hours with fresh media containing 50 µM FQI1, 50 µM FQI2-34, or 0.1% DMSO (FQI1 control) or 0.5% DMSO (FQI2-34 control). Subsequently, the cells were washed with PBS (137 mM NaCl, 2.7 mM KCl, 10 mM Na_2HPO_4, 2 mM KH_2PO_4, pH 7.2) supplemented with their respective treatments (FQI1, FQI2-34, or vehicle) and scraped from the dish in the presence of 5 mL of PBS supplemented with 1 mM Pefabloc (Sigma-Aldrich, 76307) plus the respective treatment. Pelleted cells were resuspended in 500 µL of PBS buffer with 1 mM Pefabloc plus the respective treatment. Aliquots of 50 µL of cell suspension from each treatment sample were incubated separately at the indicated temperatures for 3 minutes in a thermal cycler (Bio-rad, T100 Thermocyler), then cooled at the room temperature for 3 minutes. Lysates were prepared by four rounds of snap freezing and thawing. Soluble protein was separated from aggregates by centrifugation at 20,000 x g for 20 minutes. 40 µL of each supernatant was used for immunoblot analysis. Immunoblots were quantified with Image J and a non-parametric t-test was used to determine statistical significance.

Dual luciferase reporter assays: FQI1 and FQI34

NIH-3T3 cells were cultured at 37°C in 5% CO_2 in DMEM supplemented with 10% fetal calf serum (Atlanta Biologicals). Cells were transfected for 5 hours with the LSF-dependent reporter construct (pGL3b-4LSF WT; S4 Fig, panel B, top), the control plasmid for normalization (phRL-TK), and the expression plasmid for LSF (pEF1α-LSF), using lipofectamine (Thermo Fisher) according to the manufacturer's protocol. Vehicle (DMSO) or either FQI1 or FQI34 was then added, keeping DMSO at 0.5%. Cell extracts were harvested using 1x Passive lysis buffer (Promega Dual-Luciferase Reporter Assay System) 40-hours post-transfection. Firefly and renilla luciferase activities were measured via a dual luciferase assay (Promega). Relative luciferase activity represents firefly luciferase activity normalized to that of renilla luciferase in each extract. IC_{50} values were determined from plots of normalized luciferase activity vs. compound concentration using Prism GraphPad, non-linear regression 4-parameter curve fit with variable slope.

Generation of the DLD-1 Flp-In™ T-REx™ TIR1 BioLSF (DLD-1 derived) cell line

In order to minimize background when performing mass spectrometry of the mitotic LSF interactome, we generated a cell line in which an endogenously biotinylated fusion protein including LSF could be inducibly expressed. In order to achieve this outcome, a DNA segment encoding the biotinylated domain from the bacterial biotin carboxyl carrier protein (BCCP) was fused inframe and N-terminal to DNA encoding full-length LSF. A highly conserved family of biotin protein ligases, including endogenous enzymes spanning from bacterial to mammalian cells specifically biotinylated BCCP on one lysine within this domain[3]. In addition, the N-terminus of the BCCP-encoded cDNA was fused to DNA encoding three tandem FLAG peptide tags. The encoded fusion protein is shown in schematic in S4 Fig, panel A.

The 3XFLAG-BioLSF-encoding sequences were amplified from pCMV-3XFLAG-BioLSF and inserted into the pcDNA5/FRT/TO vector backbone (Thermo Fisher). The resulting pFRT/TO 3XFLAG-BioLSF construct was co-transfected along with the Flp recombinase-expressing plasmid (pOG44, Thermo Fisher) into DLD-1 Flp-In™ T-REx™ TIR1 cells. As per the manufacturer's instructions, cells were isolated by positive and negative selection protocols, in order to generate the DLD-1 Flp-In T-REx TIR1 BioLSF cell line, containing the specific recombination product of the introduced pFRT/TO 3XFLAG-BioLSF plasmid into the unique chromosomal FRT site in the parental DLD-1 cells. These cells constitutively express the Tet repressor, and contain Tet operator sequences in the promoter of the inserted cDNA, so that BioLSF expression is suppressed when cells are propagated in tetracycline-free media, and is induced by incubation with doxycycline (S4 Fig, panels B and C).

Characterization of BioLSF activity: Luciferase reporter assay

LSF transcriptional activity was assayed using the Dual-Luciferase® Reporter Assay System (Promega/Thermo Fisher Scientific). Assays were performed in HepG3 cells (provided by Dr. Devanand Sarkar, Virginia Commonwealth University), which were cultured in EMEM (Thermo Fisher Scientific) with 10% FBS (Atlanta Biologicals) at 37°C in 5% CO_2. Cells were co-transfected with the LSF-dependent firefly luciferase reporter construct (pGL3 4X-LSF-wt), which

[3] Reche, P.; Li, Y. L.; Fuller, C.; Eichhorn, K.; Perham, R. N. Selectivity of Post-Translational Modification in Biotinylated Proteins: The Carboxy Carrier Protein of the Acetyl-CoA Carboxylase of Escherichia Coli. *Biochem. J.* **1998**. https://doi.org/10.1042/bj3290589.

Samols, D.; Thornton, C. G.; Murtif, V. L.; Kumar, G. K.; Haase, F. C.; Wood, H. G. Evolutionary Conservation among Biotin Enzymes. *J. Biol. Chem.* **1988**. https://doi.org/10.1016/S0021-9258(18)68661-2.

contains four LSF binding sites upstream of a firefly luciferase gene[4], Renilla luciferase reporter construct (phRL-TK), which is constitutively active as an internal transfection control, and either pFRT/TO BioLSF or the pFRT/TO empty vector. Luciferase activities in cell lysates obtained two days following transfection were measured following the manufacturer's instructions. BioLSF robustly activated the reporter firefly luciferase construct (S4 Fig, panel D).

Streptavidin blotting and Immunoblotting

Proteins were separated by 7% SDS-PAGE, and transferred onto a PVDF membrane. Biotinylated proteins were detected by incubating with Streptavidin conjugated to HRP (1:15,000, Abcam ab7403). For immunoblotting, primary antibodies included mouse anti-LSF (1:1000, BD BioSciences 610818), mouse anti-β-actin (1:10,000, Sigma A1978), and mouse α-tubulin (1:1000, Fisher 62204), followed by the secondary antibody goat anti-mouse IgG-HRP (1:10,000, Thermo Fisher). After incubating with either Pierce ECL Western blotting substrate (Fisher) or Immobilon Western Chemiluminescent HRP-Substrate (Millipore), results were visualized either by exposure to X-ray film or with a Sapphire Bioimager.

[4] Grant, T. J.; Bishop, J. A.; Christadore, Bl. M.; Barot, G.; Chin, H. G.; Woodson, S.; Kavouris, J.; Siddiq, A.; Gredler, R.; Shen, X. N.; Sherman, J.; Meehan, T.; Fitzgerald, K.; Pradhan, S.; Briggs, L. A.; Andrews, W. H.; Sarkar, D.; Schaus, S. E.; Hansen, U. Antiproliferative Small-Molecule Inhibitors of Transcription Factor LSF Reveal Oncogene Addiction to LSF in Hepatocellular Carcinoma. *Proc. Natl. Acad. Sci. U. S. A.* **2012**, *109* (12), 4503–4508. https://doi.org/10.1073/pnas.1121601109.
